# Loss of PBX1 function in Leydig cells causes testicular dysgenesis and male sterility

**DOI:** 10.1007/s00018-024-05249-5

**Published:** 2024-05-09

**Authors:** Fei-Chen Wang, Xiao-Na Zhang, Shi-Xin Wu, Zhen He, Lu-Yao Zhang, Qi-En Yang

**Affiliations:** 1 Key Laboratory of Adaptation and Evolution of Plateau Biota, Northwest Institute of Plateau Biology, Chinese Academy of Sciences, Xining, 810001 Qinghai China; 2 University of Chinese Academy of Sciences, Beijing, 100049 China; 3 Qinghai Provincial Key Laboratory of Animal Ecological Genomics, Northwest Institute of Plateau Biology, Chinese Academy of Sciences, Xining, 810001 Qinghai China

**Keywords:** Leydig cell, Spermatogenesis, Testosterone, PBX1, Transcription factor

## Abstract

Leydig cells are essential components of testicular interstitial tissue and serve as a primary source of androgen in males. A functional deficiency in Leydig cells often causes severe reproductive disorders; however, the transcriptional programs underlying the fate decisions and steroidogenesis of these cells have not been fully defined. In this study, we report that the homeodomain transcription factor PBX1 is a master regulator of Leydig cell differentiation and testosterone production in mice. PBX1 was highly expressed in Leydig cells and peritubular myoid cells in the adult testis. Conditional deletion of *Pbx1* in Leydig cells caused spermatogenic defects and complete sterility. Histological examinations revealed that *Pbx1* deletion impaired testicular structure and led to disorganization of the seminiferous tubules. Single-cell RNA-seq analysis revealed that loss of *Pbx1* function affected the fate decisions of progenitor Leydig cells and altered the transcription of genes associated with testosterone synthesis in the adult testis. *Pbx1* directly regulates the transcription of genes that play important roles in steroidogenesis (*Prlr*, *Nr2f2* and *Nedd4*). Further analysis demonstrated that deletion of *Pbx1* leads to a significant decrease in testosterone levels, accompanied by increases in pregnenolone, androstenedione and luteinizing hormone. Collectively, our data revealed that PBX1 is indispensable for maintaining Leydig cell function. These findings provide insights into testicular dysgenesis and the regulation of hormone secretion in Leydig cells.

**Graphical abstract:**

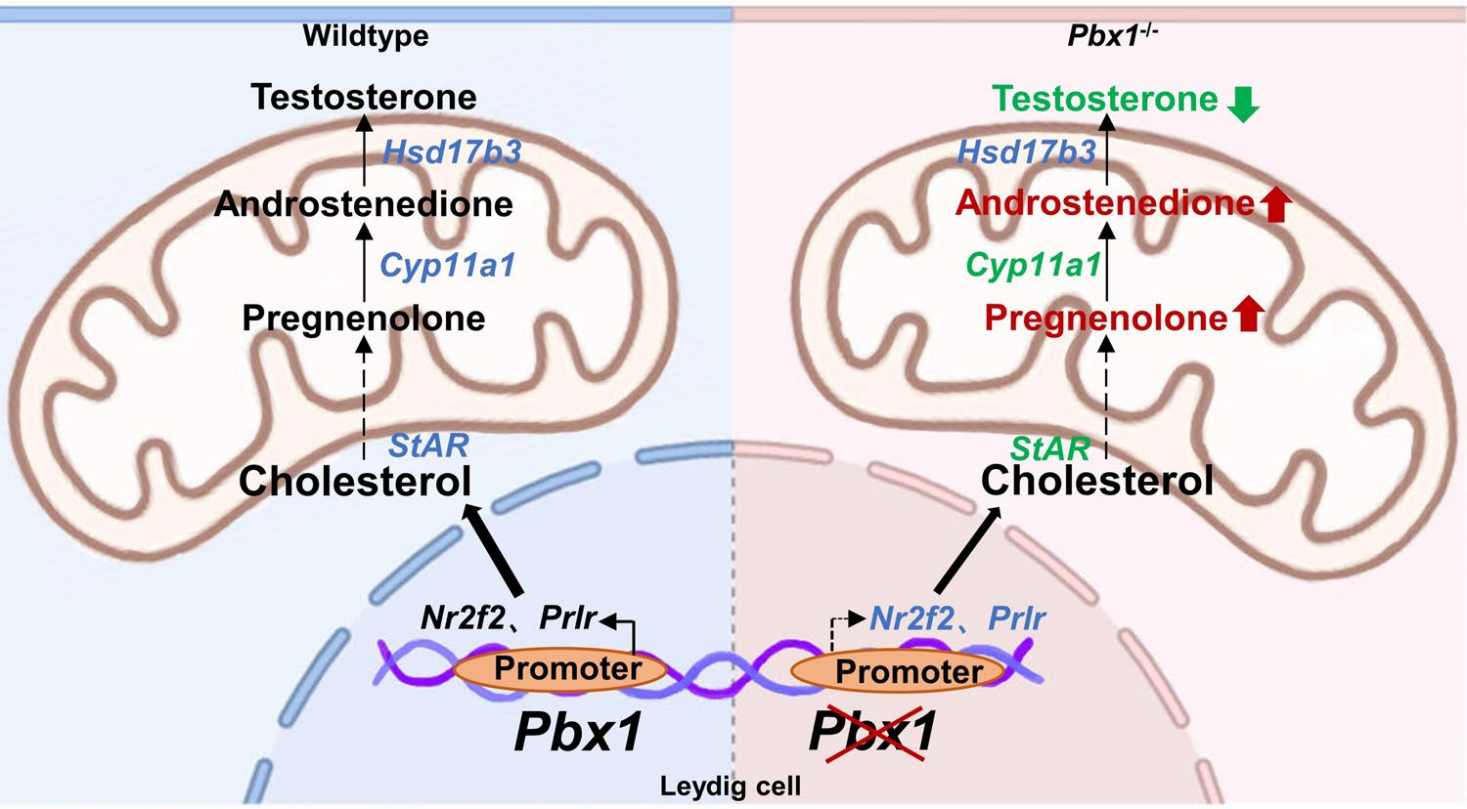

**Supplementary Information:**

The online version contains supplementary material available at 10.1007/s00018-024-05249-5.

## Introduction

Infertility is a global medical and social problem that affects 10–15% of reproductive-aged couples worldwide [1]. Male factors contribute to approximately half of these cases, and unfortunately, the number and quality of human sperm have significantly declined in the past 50 years [2]. Defective spermatogenesis is the leading cause of male infertility, and genetic factors play a major role in determining the quality of spermatogenesis [3]. Spermatogenesis is a complicated process that depends on the precise and orderly regulation of gene expression in germ cells and various somatic cells. Leydig cells are particularly important for sustaining normal spermatogenesis, and dysfunction of these cells is associated with testicular cancer [4], testicular dysgenesis [5], and azoospermia [6].

Leydig cells reside in the interstitial space of seminiferous tubules, and their major function is to produce steroid hormones. In mammals, two distinct types of androgen-producing cells are present: fetal Leydig cells (FLCs) and adult Leydig cells (ALCs) [7]. These two cell types differ in terms of their morphological characteristics, origin, and gene expression patterns [8]. FLCs are derived from multiple progenitor lineages, and androgen production by this cell population in the embryonic stage is crucial for masculinization of the male fetus [7, 9]. ALCs originate from a stem cell population that is likely established from a subset of dedifferentiated FLCs in the prepuberty testis [10, 11]. The formation of ALCs from stem Leydig cells (SLCs) involves progenitor, immature and mature states of development [12, 13]. For example, mouse SLCs initiate differentiation to produce progenitors, which become immature Leydig cells under the induction of insulin-like growth factor (IGF-1) at postnatal day (PD) 29–35. Immured cells expand in number and differentiate to become functional ALCs around PD49 [14]. Once ALCs are formed, they remain quiescent and rarely die under undisturbed conditions during adulthood; however, if the original ALCs are eliminated experimentally, new cells are generated [15, 16]. Approximately 95% of sex hormones are secreted by ALCs under the control of the hypothalamic–pituitary–gonadal (HPG) axis.

Genetic evidence reveals essential roles of testosterone in multiple developmental events in spermatogenesis. Testosterone synthesis relies on the activities of at least four steroidogenic enzymes in Leydig cells, including cytochrome P450 cholesterol side chain cleavage enzyme (CYP11A1), 3β-hydroxysteroid dehydrogenase (3β-HSD), cytochrome P450 17α-hydroxylase/17,20-lyase (CYP17A1) and 17β-hydroxysteroid dehydrogenase isoform 3 (17β-HSD3) [17]. Cholesterol is transported into mitochondria by STAR and converted into pregnenolone by CYP11A1 [18]. Pregnenolone diffuses into the smooth endoplasmic reticulum and is converted to progesterone by dehydrogenation via a 3β-HSD-dependent mechanism. Progesterone is then catalyzed by 17α-hydroxylase to hydroxyprogesterone, which is further catalyzed by C17,20-lyase to androstenedione. Finally, testosterone is converted from androstenedione by the action of 17β-hydroxysteroid dehydrogenase in a reversible reaction [19]. Notably, FLCs cannot produce testosterone in rodents because of the lack of expression of 17β-HSD3 [13]. In the testis, testosterone binds to its receptor (androgen receptor, Ar) on Sertoli cells, peritubular myoid cells (PTMCs) and Leydig cells to direct essentially all phases of spermatogenesis in a cell content-dependent manner [20]. For example, androgen signaling mediated through Sertoli cells is needed for meiosis and terminal differentiation of spermatids, while its actions on PTMCs support the development of spermatids and maintenance of spermatogonial stem cells (SSCs) [21–23]. Testosterone deficiency leads to male sexual dysfunction, decreased reproductive capacity, and primary or delayed hypogonadism [24]. Defective testosterone production is also associated with other diseases, such as cardiovascular disease [25] and diabetes [26]. However, the molecular signaling pathways that determine Leydig cell differentiation and testosterone synthesis remain unclear.

The transcription factor pre-B-cell leukemia homeobox 1 (PBX1) is pivotal for embryogenesis, organogenesis, and development. PBX1 was originally identified as a part of a fusion protein produced by chromosomal translocation in pre-B-cell acute lymphoblastic leukemia [27]. Genetic ablation of *Pbx1* in mice causes embryonic lethality due to defects in renal hypoplasia, endocrine cell differentiation and skeletal patterning [28, 29]. PBX1 is also indispensable for proper differentiation of the urogenital system; specifically, it appears to be essential for fetal Leydig cell development [28]. A PBX1 missense mutation is associated with gonadal dysgenesis in humans [30]. In the postnatal murine testis, PBX1 is found in Leydig cells, peritubular myoid cells and Sertoli cells [31]. Despite these findings, the roles of PBX1-dependent transcription networks in spermatogenesis have not been fully determined.

In the present study, we investigated the expression pattern of PBX1 in the testis and evaluated its functional role in spermatogenesis using a conditional knockout approach. We showed that PBX1 was highly expressed in Leydig cells and peritubular myoid cells in adult testes. Conditional deletion of *Pbx1* in Leydig cells caused spermatogenic defects and complete sterility. *Pbx1* directly regulates the expression of *Prlr*, *Nr2f2* and *Nedd4* to direct testosterone biosynthesis and Leydig cell differentiation. This study provides a novel  molecular mechanism underlying the fate determinations and hormone secretion of mouse Leydig cells.

## Results

### Expression and localization of PBX1 in the mouse testis at different stages

**Fig. 1:**
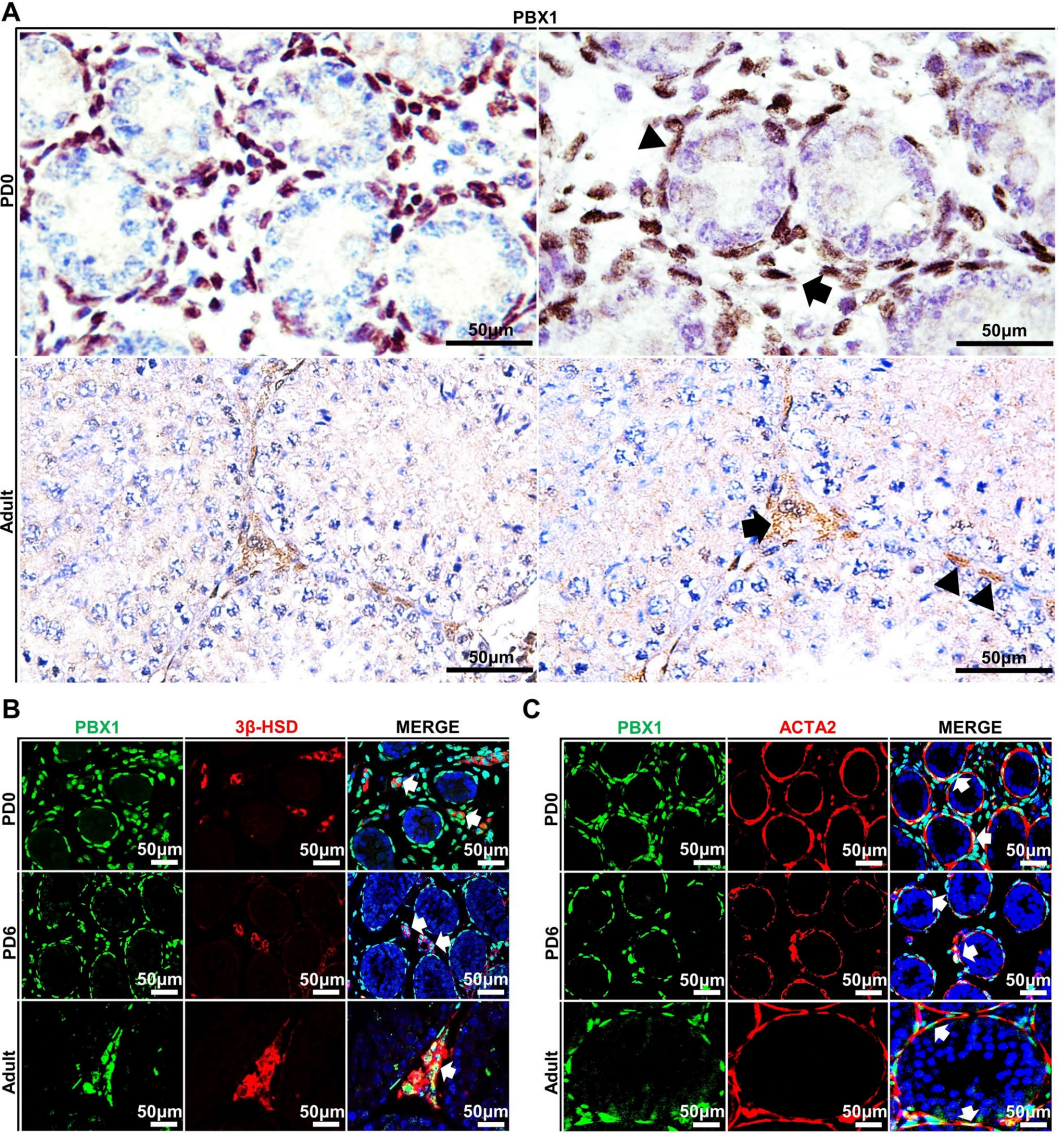
PBX1 expression was restricted to Leydig cells and peritubular myoid cells in mice. **A** Immunohistochemical staining of PBX1 in testicular cross-sections of wild-type mice at postnatal days (PDs) 0 and 60 (adult). Scale bar = 50 µm. The arrow indicates LCs, and the triangle indicates peritubular myoid cells. **B** Immunofluorescence staining of 3β-HSD (red) and PBX1 (green) in testicular cross-sections of wild-type mice at PD0, PD6 and PD60. **C** Immunofluorescence staining of ACTA2 (red) and PBX1 (green) in testicular cross-sections of wild-type mice at PD0, PD6 and adult. Scale bar = 50 µm. Three biological replicates were conducted for each experiment (*n* = 3)

To determine the expression and cellular localization of PBX1 in the testis, we conducted immunohistochemical (IHC) and immunofluorescent (IF) staining on cross-sections of mouse testicular tissues at different postnatal days (PD) 0, 6 and 60. The results confirmed that an immunoreactive signal for the PBX1 antibody was detected only in the Leydig cells and peritubular myoid cells (PTMCs) of the testes from neonatal and adult mice (Fig. 1A). No signal was present in the negative control (Supplementary Fig. 1A). Costaining of PBX1 with the Leydig cell marker 3β-HSD and PTMCs marker ACTA2 revealed that Leydig cells and PTMCs both expressed PBX1 at these three stages (Fig. 1B, C, Supplementary Fig. 1B). We did not observe the expression of PBX1 in Sertoli cells of the PD0 or PD6 testes. Moreover, PBX1 protein was not detectable in spermatogenic cells of PD0, PD6 and PD60 testes. (Supplementary Fig. 3A, B). In the ovaries of female mice, PBX1 was ubiquitously expressed in oocytes and somatic cells (Supplementary Fig. 2A, B). These data suggested that Leydig cells and PTMCs from neonatal and adult testes expressed PBX1. Because Leydig cells and PTMCs share common progenitors during fate specification [32], these two closely related somatic lineages activate the expression of PBX1 in the fetal testis and sustain its expression during postnatal development.

### Conditional deletion of *Pbx1* in Leydig cells leads to testicular atrophy and sterility

**Fig. 2:**
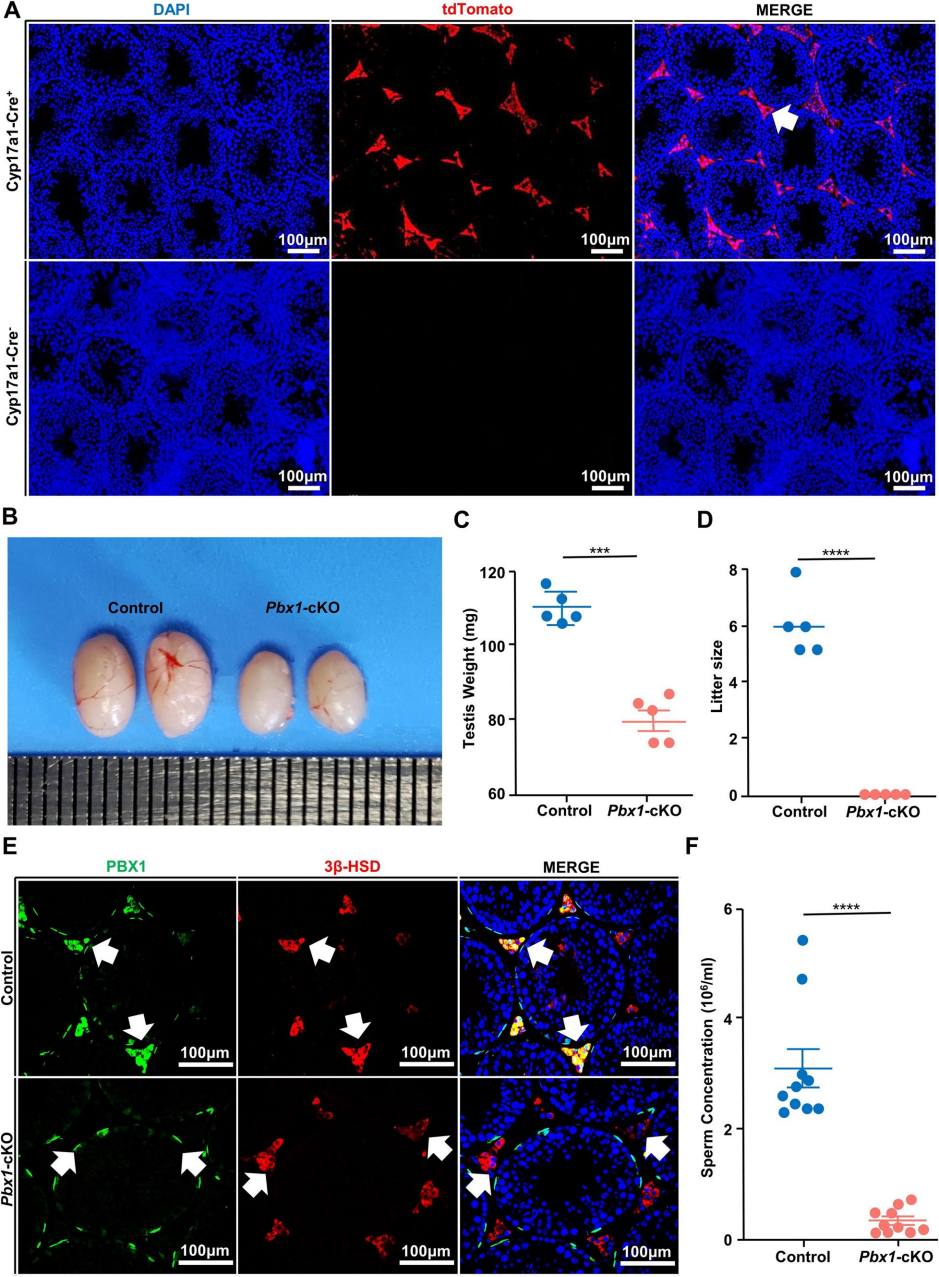
*Pbx1* deletion in Leydig cells caused testicular atrophy and sterility. **A** Immunofluorescence staining for tdTomato (red) and DAPI (blue) in cross-sections of testes from *Cyp17a1-iCre*^*[KI/*+*]*^; *Rosa26-LSL-tdTomato*^*[CKI/*+*]*^ and control testes (*n* = 3). Scale bar = 100 µm. **B** Representative images of testes from 4-month-old control and *Pbx1*-cKO male mice. **C** Testis weight (mg) of 4-month-old control (*n* = 5) and *Pbx1*-cKO (*n* = 5) mice. **D** Litter sizes of control and *Pbx1*-cKO male mice after mating with wild-type females. *Pbx1*-cKO males were completely sterile. Five control and *Pbx1*-cKO male mice were used for fertility assessment (*n* = 5). **E** Immunofluorescence staining for 3β-HSD (red) and PBX1 (green) in cross-sections of testes from control and *Pbx1*-cKO male mice (*n* = 3). The white arrow indicates 3β-HSD^+^ Leydig cells. Scale bar = 100 µm. **F** Quantification of sperm concentrations in 4-month-old control and *Pbx1*-cKO mice (*n* = 10). *** and **** denote significant differences at *P* < 0.001 and *P* < 0.0001, respectively. The error bars represent the SDs

Given its significant roles in testosterone production and spermatogenesis, we next asked whether Leydig cells rely on PBX1 activity by conditionally deleting *Pbx1* in mouse Leydig cells using the Cre–LoxP system (Supplementary Fig. 4A). We crossed *Pbx1*^*flox/flox*^* (Pbx1*^*fl/fl*^*)* mice with the *CYP17a1-Cre* line, which expresses Cre recombinases in Leydig cells at embryonic day (E12.5) [33]. The *CYP17a1-Cre; Pbx1*^*fl/fl*^ (hereafter referred to as *Pbx1*-cKO) and littermate control (*CYP17a1-Cre; Pbx1*^*fl/*+^) mice were genotyped, and testis sections were collected for histological analysis (Supplementary Fig. 4A, B). *Cyp17a1-iCre* mice were mated with Rosa26-LSL-tdTomato mice to produce double-gene heterozygous mice. The expression of the tdTomato protein in the testes of 6-week-old mice was detected to determine the activity of the Cre recombinant enzyme. *Cre*^+^ (*Cyp17a1-iCre *^*[KI/*+*]*^; *Rosa26-LSL-tdTomato *^*[CKI/*+*]*^) mice emitted a large amount of red fluorescence from the tdTomato protein in Leydig cells, while no tdTomato signal was detected in the testes of control group (*Cre*^*−*^) mice, indicating the reliability of the *CYP17a1-Cre-*mediated recombination (Fig. 2A).

We then examined the impact of conditional *Pbx1* knockout on testis development and spermatogenesis. The body weight of adult *Pbx1*-cKO animals was not different from that of control animals; however, the testis weight of *Pbx1*-cKO mice was significantly lower (79.76 ± 2.72 vs 109.90 ± 1.99 mg control, *n* = 5) (Fig. 2B, C). The results of the fertility assessment revealed a complete sterile phenotype in 4-month-old *Pbx1*-cKO males (Fig. 2D). The control animals sired an average of 6 ± 1.22 pups, but the *Pbx1*-cKO animals did not sire any pups (*n* = 5), indicating complete sterility. The immunostaining results revealed that *Pbx1* was eliminated in Leydig cells and that its presence in peritubular myoid cells was not impacted, thus demonstrating that *Pbx1* was successfully deleted in only Leydig cells (Fig. 2E). The knockout efficiency was 87.29% ± 0.73 (*n* = 3). The sperm concentration was reduced by 89.71% compared with that of control mice (3.08 ± 0.34 vs 0.32 ± 0.07 × 10^6^/mL control, *n* = 10) (Fig. 2F). *Pbx1* is suggested to play important roles in regulating estrogen production [34]; therefore, we utilized the same approach to obtain *Pbx1*-cKO female mice and examined the phenotype of the females (Supplementary Fig. 4C). *Pbx1*-cKO female mice were healthy and fertile (Supplementary Fig. 4E). Moreover, there was no significant difference in ovary histology between the control and *Pbx1*-cKO animals (Supplementary Fig. 4D, F). The proportions of preantral and antral follicles did not differ between control and *Pbx1*-cKO females (Supplementary Fig. 4G). Taken together, these data demonstrated that PBX1 in Leydig cells was essential for sustaining normal fertility in mice.

### *Pbx1* deletion in Leydig cells altered seminiferous tubule structure

**Fig. 3:**
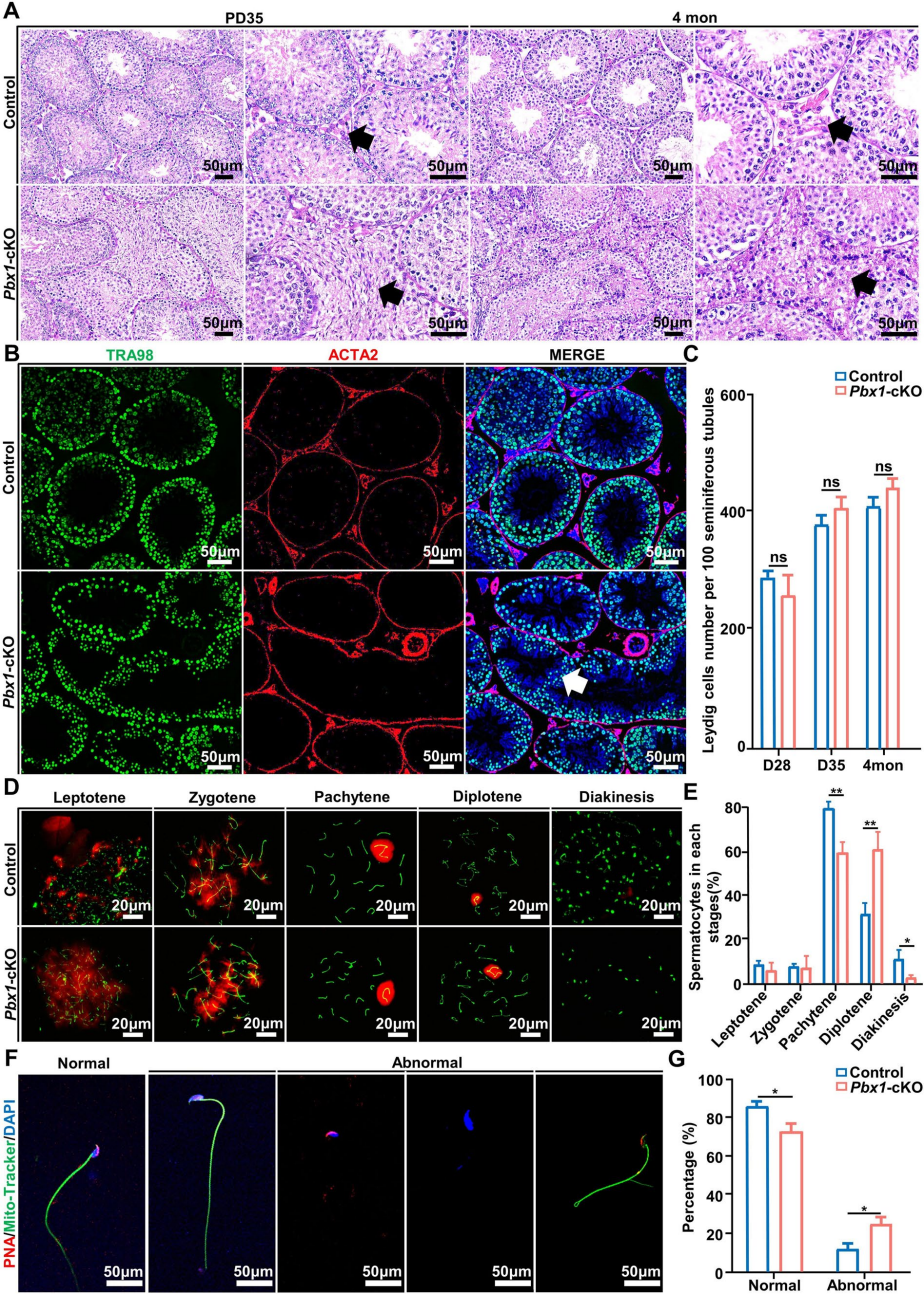
*Pbx1* deletion in Leydig cells disrupted the integrity of the seminiferous tubules and led to defects in spermatocytes and sperm cells. **A** H&E-stained testes of control and *Pbx1*-cKO mice at PD35 and PD120. The black arrow indicates the interstitial space of the seminiferous tubules. Germ cells enter interstitial area in the *Pbx1*-cKO mice (*n* = 3 for each experiment). Scale bar = 50 µm. **B** Immunofluorescence staining of ACTA2 (red) and TRA98 (green) in cross-sections of testes from 4-month-old mice (*n* = 3). Scale bar = 50 µm. **C** Leydig cell numbers in 100 seminiferous tubules from control and *Pbx1*-cKO mice at PD28, PD35 and 4 months of age (*n* = 5). **D** Chromosome spreading and immunofluorescence staining for SYCP3 (green) and γH2AX (red) in spermatocytes from testes of control and *Pbx1*-cKO male mice. Scale bar = 20 µm. **E** Proportion of spermatocytes at leptotene, zygotene, pachytene, diplotene and diakinesis stages of meiosis from control and *Pbx1*-cKO male mice (*n* = 3). **F** Immunofluorescence staining of the acrosome (red) and mitochondria (green) in epididymal sperm of control and *Pbx1*-cKO animals, which revealed morphological differences between normal and abnormal sperm. **G** Quantification of sperm malformation rates of 4-month-old control and *Pbx1*-cKO mice (*n* = 3). Scale bar = 50 µm. * and ** denote significant differences at *P* < 0.05 and *P* < 0.01; ns denotes not significant, respectively. The error bars represent the SDs

Although a marked decrease in the sperm concentration was evident, this change might not be the sole reason for the sterile phenotype of the knockout mice. We next conducted histological analysis to investigate the changes in the seminiferous tubules induced by *Pbx1* deletion in Leydig cells. To this end, we first performed hematoxylin–eosin (H&E) staining of testicular cross-sections from control and knockout animals at the neonatal, prepubertal and adult stages. In 4-month-old *Pbx1*-cKO mice, seminiferous tubules were profoundly disrupted, and disorganized spermatogenic cells appeared in the interstitial tissue of the testis (Fig. 3A). The percentage of tubules containing abnormal spermatogenic tubules reached 74% (*n* = 3). Immunostaining for the germ cell marker gene TRA98 confirmed that spermatogenic cells were abundantly present in the testicular interstitial space, likely due to damage to the basement membrane (Fig. 3B). We then examined the seminiferous tubules from PD0, PD14 and PD28 to determine the developmental stages at which the effect of *Pbx1* deletion began to emerge. The results showed that the integrity of the seminiferous tubules was intact in the neonatal testis (Supplementary Fig. 5A). Abnormalities in seminiferous tubules and the organization of spermatogenic cells were first detected in the testis at PD14, and the number reached 70% at PD28 (*n* = 3). We counted the number of 3β-HSD^+^ Leydig cells in 100 round seminiferous tubules at PD28, PD35 and 4 months of age. Although the expression of 3β-HSD was significantly reduced by *Pbx1* deletion, the total population of Leydig cells from the testes of control and *Pbx1*-cKO mice did not significantly differ at PD35 or 4 months of age (*n* = 5) (Fig. 3C). Collectively, these data indicated that the loss of *Pbx1* in Leydig cells resulted in disorganization of seminiferous tubules and damage to the testicular microenvironment.

### Development of meiotic and postmeiotic cells was impacted by the loss of *Pbx1* in Leydig cells

Spermatogenesis includes three phases: mitosis of spermatogonia, meiosis of spermatocytes and spermiogenesis. The homeostasis of undifferentiated spermatogonia was not affected by *Pbx1* knockout because the number of LIN28^+^ spermatogonia per 500 Sertoli cells was similar between control and *Pbx1-*cKO animals (Supplementary Fig. 5B). The distribution and extent of DNA double-strand breaks (DSBs) marked by γH2AX were not affected by *Pbx1* knockout; however, chromosome spreading showed that the proportions of cells in the leptotene, zygotene, pachytene, diplotene, and diakinesis stages were altered (Fig. 3D), indicating that spermatocyte progression was mildly impacted in *Pbx1*-cKO mice. Specifically, the proportion of pachytene spermatocytes decreased (23.18 ± 2.33 vs 44.62 ± 3.92 control, *n* = 3), while the diplotene spermatocyte proportion increased (58.73 ± 1.31 vs 43.92 ± 2.47 control, *n* = 3) (Fig. 3E). Collectively, these data showed that the meiotic progression of spermatocytes was affected by *Pbx1* knockout.

Next, we examined sperm abnormalities, including abnormal distribution of mouse sperm mitochondria, defects in nuclear structure, and defects in sperm flagella, as described previously [35, 36]. Spermatozoa were collected from the epididymis of 4-month-old control and *Pbx1*-cKO mice and were stained for the acrosome (PNA) and mitochondria (MitoTracker). The results showed that the percentage of abnormal sperm in *Pbx1*-cKO mice was significantly greater than that in control mice (11.88 ± 1.85 vs 24.69 ± 2.55 control, *n* = 3) (Fig. 3F, G). Taken together, these results suggest that the absence of *Pbx1* in Leydig cells impacts meiosis and spermatid morphology.

### Loss of *Pbx1* altered the gene expression profiles of Leydig cells at the single-cell level

**Fig. 4:**
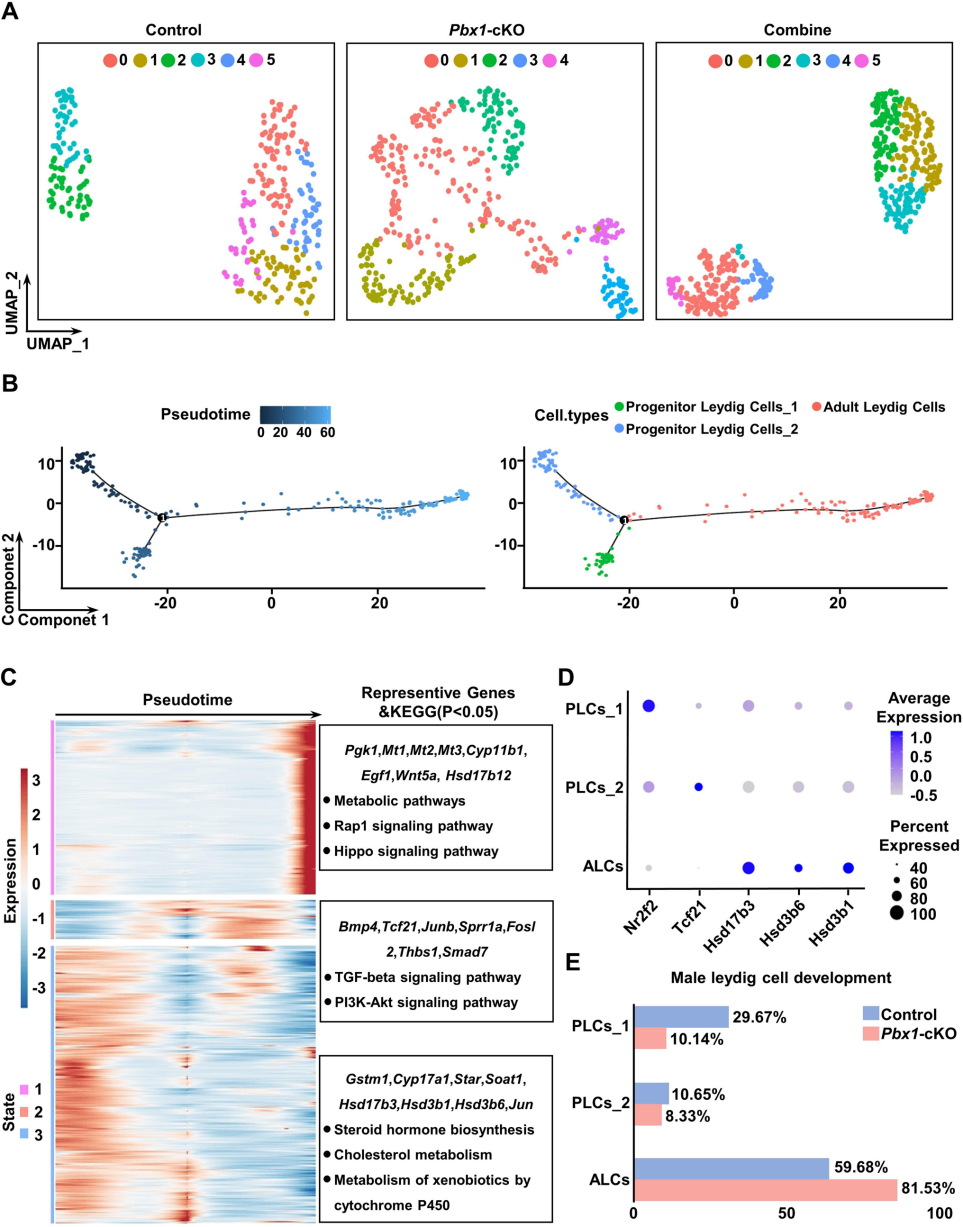
Gene expression was altered in Leydig cells from *Pbx1*-cKO mice at single-cell level. **A** Dimensionality reduction clustering at a resolution = 1.2 using Uniform Manifold Approximation and Projection (UMAP). **B** Pseudotime trajectories and cell types according to UMAP from the integrated cells from control and *Pbx1*-cKO mice. **C** Heatmap of branch points corresponding to the pseudotime axis of integrated Leydig cells and representative genes and KEGG pathways in each state. Three different states were identified. **D** Dot plot for the expression of selected marker genes (*Nr2f2*, *Tcf21*, *Hsd17b3*, *Hsd3b6* and *Hsd3b1*) across Leydig cell types in control and *Pbx1*-cKO mice. **E** The percentages of progenitor Leydig cells (1&2) and adult Leydig cells in control and *Pbx1*-cKO mice based on scRNA-seq analysis

Single-cell RNA-seq (scRNA-seq) is a powerful tool for depicting developmental trajectory and gene expression in the testis; thus, we next examined the patterns and dynamics of gene expression within different Leydig cell subpopulations in the testes of control and *Pbx1*-cKO mice. Testicular single cells from 2 control and 2 *Pbx1*-cKO mice were isolated and processed for scRNA-seq analysis using the BD Rhapsody™ Single-Cell System [37]. On average, 7450 and 7439 genes; 27,607 and 26,887 unique molecular indexes (UMIs); and 2.61% and 2.88% mitochondrial percentages were detected per library for the control and *Pbx1*-cKO testes, respectively (Supplementary Fig. 6A). Leydig cells were extracted for the expression of marker genes (*Star*, *Nr2f2*, *Tcf21*, *Hsd3b1*, *Hsd17b3*, *Cyp11a1*, *Cyp17a1* and *Hsd3b6*) for clustering and detection of all differentially expressed genes (DEGs). As a result, 310 and 444 Leydig cells were obtained from control and *Pbx1*-cKO mice, respectively. *Pbx1* was widely expressed in all the control Leydig cells but was not expressed in the cells from the *Pbx1*-cKO testes (Supplementary Fig. 6B). Uniform Manifold Approximation and Projection (UMAP) analysis identified 6 clusters for the control cells and 5 clusters for the *Pbx1*-cKO Leydig cells at a resolution of 1.2 (Fig. 4A); these cell clusters were classified into three cell types, namely, progenitor Leydig cells_1 (PLCs_1), progenitor Leydig cells_2 (PLCs_2) and adult Leydig cells (ALCs) (Supplementary Fig. 6C). The integrated Leydig cells had one branch point according to pseudotime and were divided into three states; moreover, the developmental trajectory showed that ALCs originated from two different sources (Fig. 4B). Gene expression patterns varied among different Leydig cell subpopulations. For example, *Nr2f2* was specific to PLCs_1, *Tcf21* was enriched in PLCs_2, and *Hsd17b3*, *Hsd3b6,* and *Hsd3b1* were abundantly present in ALCs (Fig. 4C, D). By counting the proportions of cells in different states, it was discovered that loss of *Pbx1* function changed the developmental dynamics of Leydig cells through a decrease in the number of progenitor cells. For example, the proportion of PLCs_1 decreased by 19.53%, and that of PLCs_2 decreased by 2.32%. Importantly, the percentage of ALCs increased by 21.85% (Fig. 4E). Therefore, these data indicated that the stem Leydig cell pool was severely affected and that the ALC population was expanded upon *Pbx1* ablation.

**Fig. 5:**
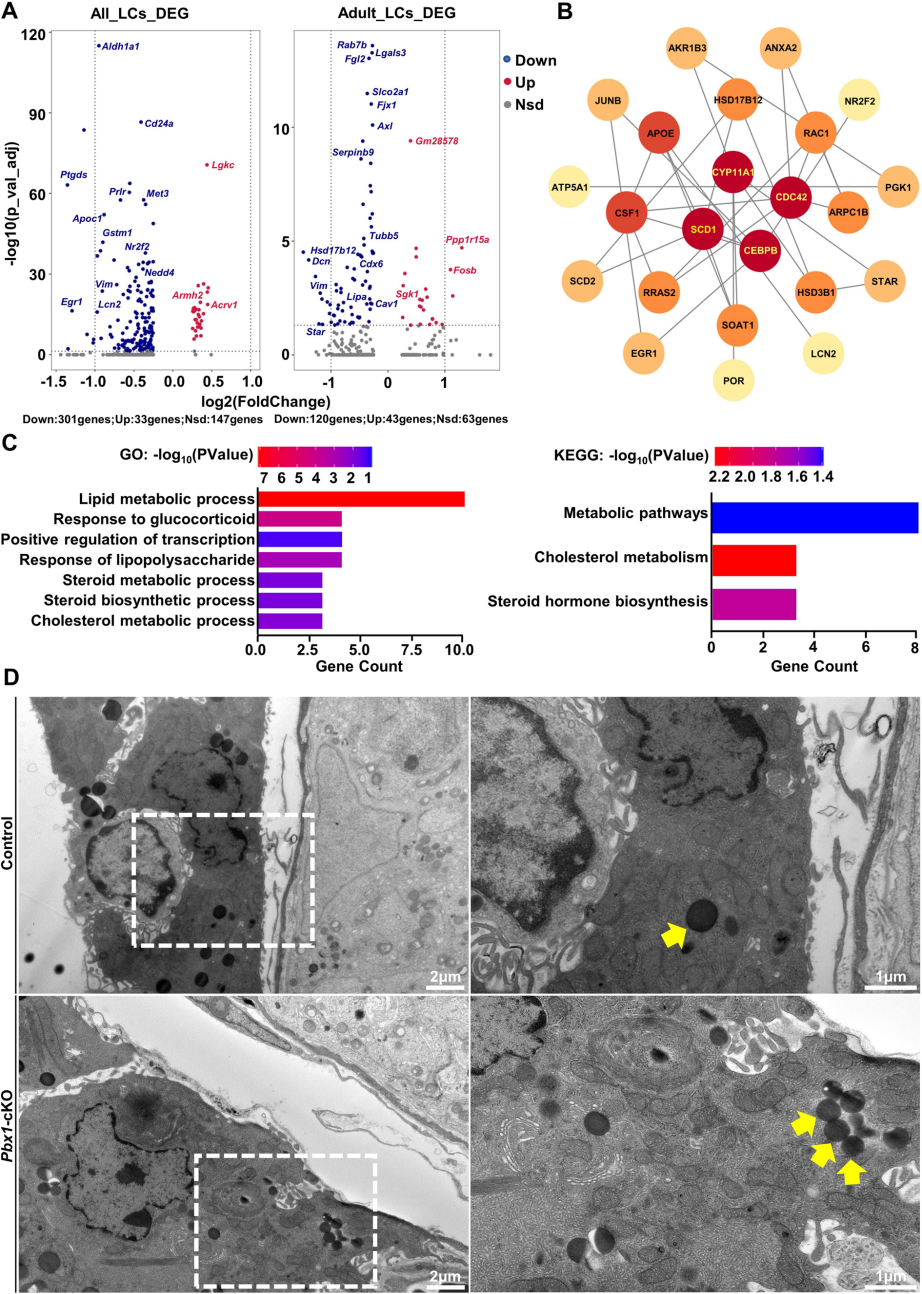
*Pbx1* directs the expression of genes involved in steroidogenesis and Leydig cell development. **A** Volcano plot of genes differentially expressed in all types of Leydig cells (left panel) and in adult Leydig cells (right panel) between *Pbx1*-cKO and control mice. The *X* axis fold change and the *Y* axis is adjusted *P* value. **B** The protein interaction network of the DEGs was visualized with Cytoscape software. **C** GO enrichment and KEGG pathway analyses of DEGs between control and *Pbx1*-cKO Leydig cells. Lipid metabolism was significantly impacted by *Pbx1* deletion in Leydig cells. **D** Representative transmission electron microscopy images of Leydig cells from control and *Pbx1*-cKO testes. The yellow arrow indicates lipid droplets. Scale bar = 2 µm (× 8000), scale bar = 1 µm (× 20,000)

Next, we analyzed the differentially expressed genes (DEGs) between *Pbx1*-cKO and control Leydig cells according to cell type using the Findmarkers function in Seurat. A total of 334 DEGs were identified, 90.12% of which (301/334) were downregulated in *Pbx1* knockout Leydig cells, indicating that the major function of *Pbx1* is to promote gene expression (Fig. 5A, Supplementary Table 2). We also extracted ALCs and found that the expression of 163 genes was altered by *Pbx1* deletion. We found that 73.62% of the genes (120/163) were downregulated and that 26.38% (43/163) of the genes were upregulated (Fig. 5A, Supplementary Table 3). STRING was used to construct differential gene interaction networks, which were visualized using Cytoscape (Fig. 5B). These analyses indicated that *Pbx1* may directly target *Nr2f2* and other genes involved in steroid biogenesis and Leydig cell differentiation. GO enrichment and KEGG analyses revealed that lipid metabolic processes and pathways involved in cholesterol metabolism, metabolic pathways, and steroid hormone biosynthesis were potentially affected by these DEGs (Fig. 5C, Supplementary Table 4). For example, *Prlr*, *Hsd17b12, Star*, *Nr2f2*, *Egr1*, *Mt1*, *Lcn2*, *Cyp11a1*, *Vim*, *Apoc1* and *Nedd4* were significantly impacted by the loss of *Pbx1* function. We then examined the ultrastructure of Leydig cells in the testes of control and *Pbx1*-cKO mice using transmission electron microscopy (TEM). The morphology and distribution of lipid droplets were dramatically different between the two different genotypes (Fig. 5D). When the number of lipid droplets increased in the *Pbx1*-cKO testes, the size of the droplets decreased significantly (0.21 ± 0.02 vs 0.56 ± 0.02 µm control) (Fig. 5D). In summary, the results of these analyses revealed that *Pbx1* deletion in Leydig cells resulted in defects in lipid metabolism and steroidogenesis.

**Fig. 6:**
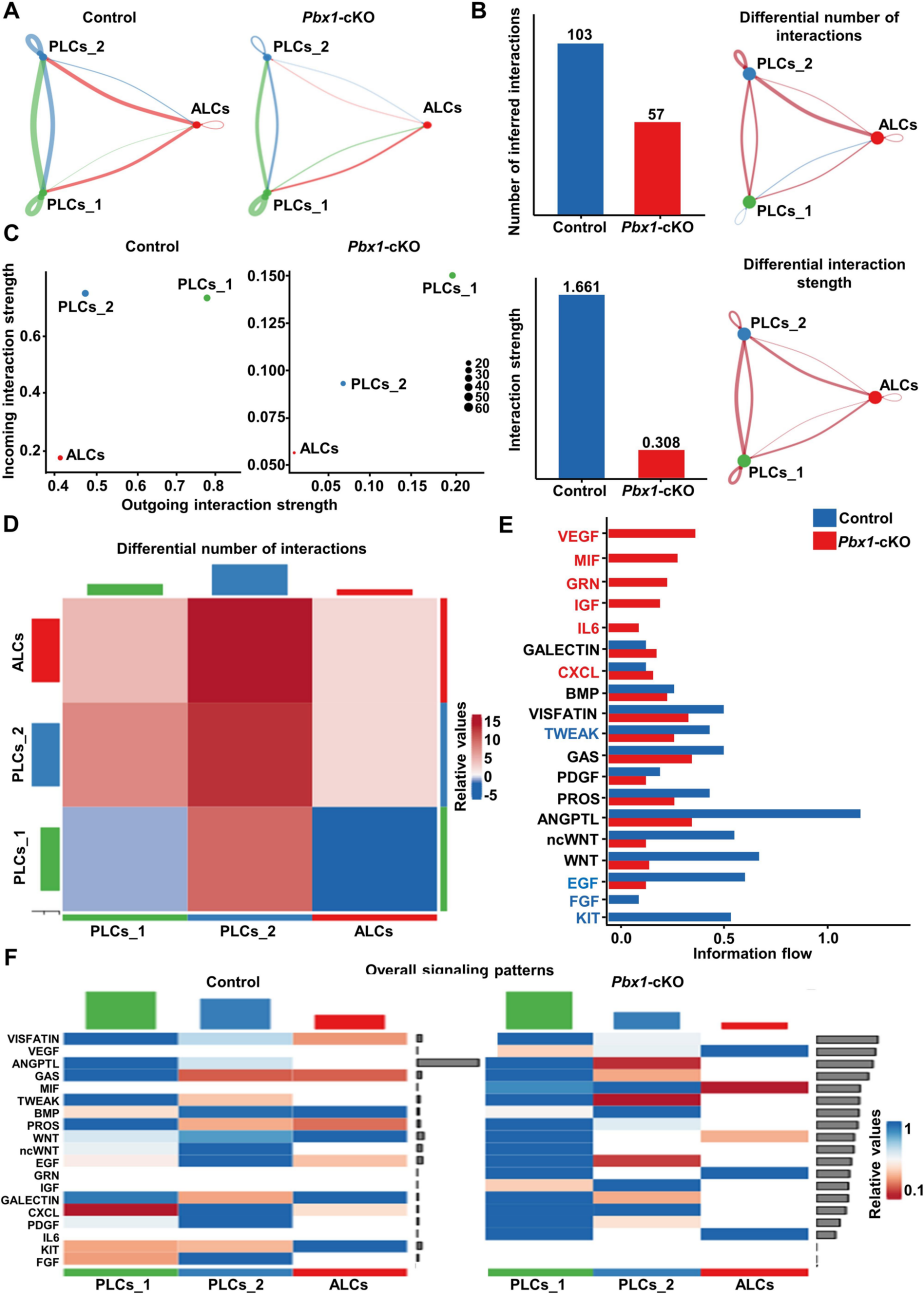
Cell-to-cell communication between different types of Leydig cells decreased after *Pbx1* deletion, and the interactions were significantly altered. **A** Number and intensity of interactions between two cell populations in each sample. **B** Comparison of the number and strength of interactions in the cell‒cell communication network between control and *Pbx1*-deficient Leydig cells. **C** Comparison of the strength of outgoing and incoming interactions in 2D space between control and *Pbx1*-deficient Leydig cells. **D** Heatmap of the number and strength of interactions between control and *Pbx1*-deficient Leydig cells. **E** Identification and visualization of conserved and specific signaling pathways. **F** Comparison of overall signaling patterns associated with each cell population

Based on the above results, we noticed that Leydig cell populations responded to *Pbx1* deletion in a cell context-dependent manner. We next used the CellChat package to examine the probability of communication between PLCs_1 or PLCs_2 and ALCs. The results showed that the number of ligand-receptor pairs between PLCs_1 and PLCs_2 and ALCs was reduced in the *Pbx1-*cKO samples (Fig. 6A). We then examined the differences in the number and intensity of cell-to-cell communication and found that the number of *Pbx1*-deficient Leydig cells (57 vs 103 controls) and the communication intensity decreased substantially (0.308 vs 1.661 controls) (Fig. 6B). By comparing the intensity of afferent and efferent interactions between the control and *Pbx1*-cKO cells, it was observed that the intensity of incoming and outgoing interactions of the PLCs_1, PLCs_2 and ALCs decreased (Fig. 6C, D). For example, the VEGF, MIF, GRN, IGF and IL6 signaling pathways were enhanced, but FGF and KIT signaling were diminished in cells from *Pbx1*-cKO animals (Fig. 6E, F). Signaling mediated through VISDATIN, PDGF, ANGPTL1 and EGF was also different in Leydig cells from control and *Pbx1*-cKO animals (Supplementary Fig. 7). Taken together, these data showed that *Pbx1* deletion in Leydig cells changed the cell-to-cell communication between different subpopulations of Leydig cells.

**Fig. 7:**
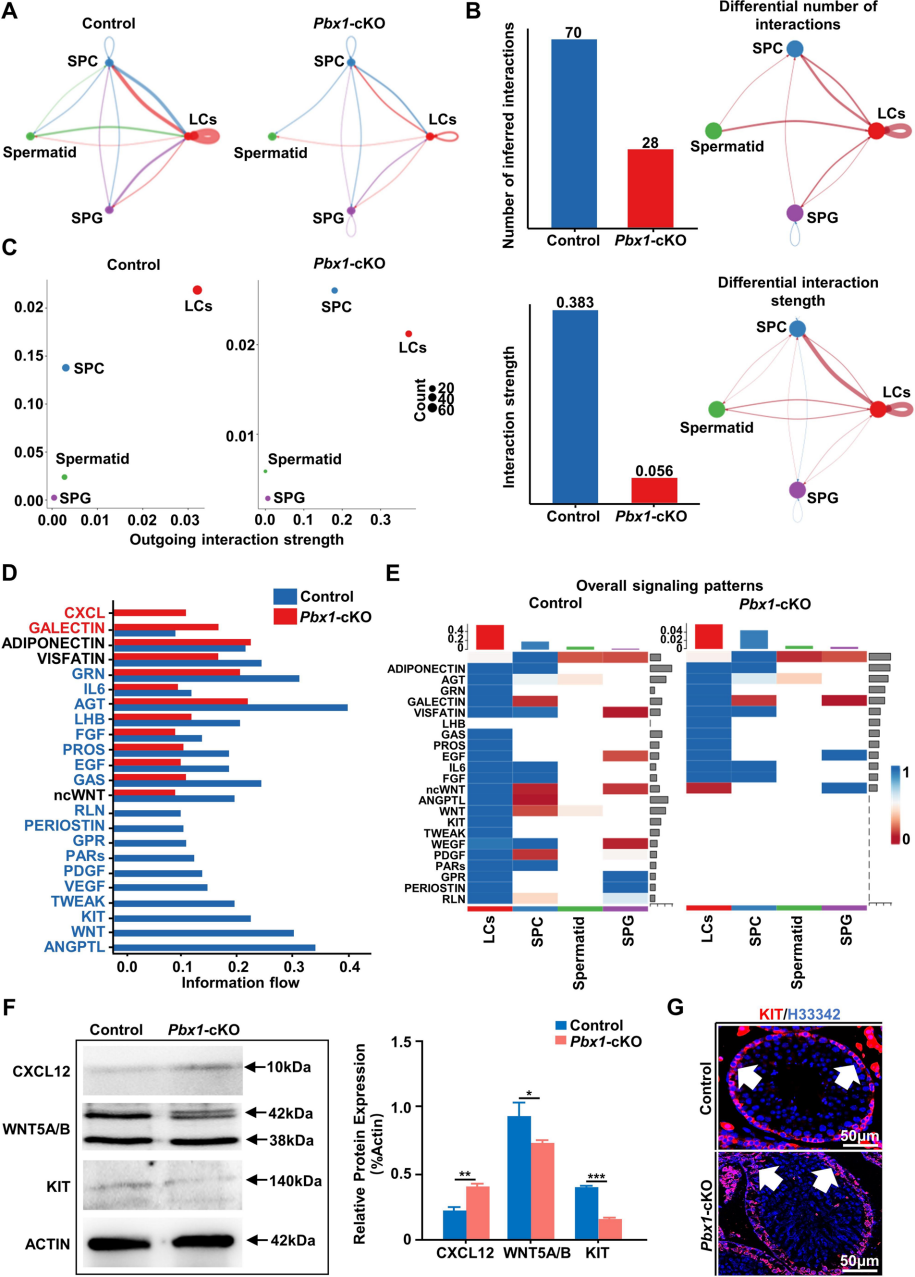
Cell-to-cell communication between Leydig cells and different types of germ cells, especially spermatocytes, is significantly altered after *Pbx1* deletion. **A** Number and intensity of interactions between Leydig cells and germ cells (including spermatogonia, spermatocytes and spermatids) in each sample. **B** Comparison of the number and strength of interactions in the cell‒cell communication network between control and *Pbx1*-deficient samples. **C** Comparison of efferent and afferent interaction strengths in 2D space was used to identify cell populations whose signals were significantly different between the control and *Pbx1*-cKO samples. **D** Comparison of the strength of the information flow through each signaling pathway revealed changes in the signaling pathway between the control and *Pbx1*-cKO samples. **E** Comparison of overall signaling patterns associated with each cell population. **F** Representative western blot images showing CXCL12, WNT5A/B and KIT expression in control and *Pbx1*-cKO testes (*n* = 3). The protein expression levels were calculated relative to those of beta-actin. **G** Immunofluorescence staining of cKIT (red) of testes from adult mice (*n* = 3). Scale bar = 50 µm

We further analyzed the changes in cellular communication between germ cells and Leydig cells. The number of ligand-receptor pairs between germ cells (including spermatogonium, spermatocyte and spermatid cells) and Leydig cells was reduced in the *Pbx1-*cKO samples (Fig. 7A). We then examined the differences in the number and intensity of cells engaged in cell-to-cell communication and found that the number of *Pbx1*-deficient Leydig cells (28 vs 70 controls) and the communication intensity (0.056 vs 0.383 controls) decreased substantially (Fig. 7B). After the loss of *Pbx1*, the cell population that sends or receives signals also changes (Fig. 7C). By comparing the information flow of each signaling pathway to identify conservative and context-specific signaling pathways, we found that the CXCL, GALECTIN and ADIPONECTIN signaling pathways were enhanced, but most signaling pathways, such as the EGF, FGF, KIT, WNT and PDGF signaling pathways, decreased, or even disappeared (Fig. 7D). Moreover, the expression of genes associated with signal transduction pathways, especially the WNT, KIT, TWEAK, WEGF, PDGF and other signaling pathways, also changed (Fig. 7E). Finally, the expression level of CXCL12, WNT5 A/B and KIT were verified by western blotting, the results confirmed that the expression of CXCL12 increased significantly while the relative levels of WNT5A/B and KIT significantly decreased in the testis of *Pbx1*-cKO mice (Fig. 7F). Results of immunofluorescence staining confirmed that KIT expression decreased in spermatogenic cells (Fig. 7G). These data showed that cell-to-cell communication between germ cells and Leydig cells was altered, especially between Leydig cells and spermatocytes.

### ChIP-seq reveals that ***Pbx1*** directly targets genes involved in steroid hormone synthesis

**Fig. 8:**
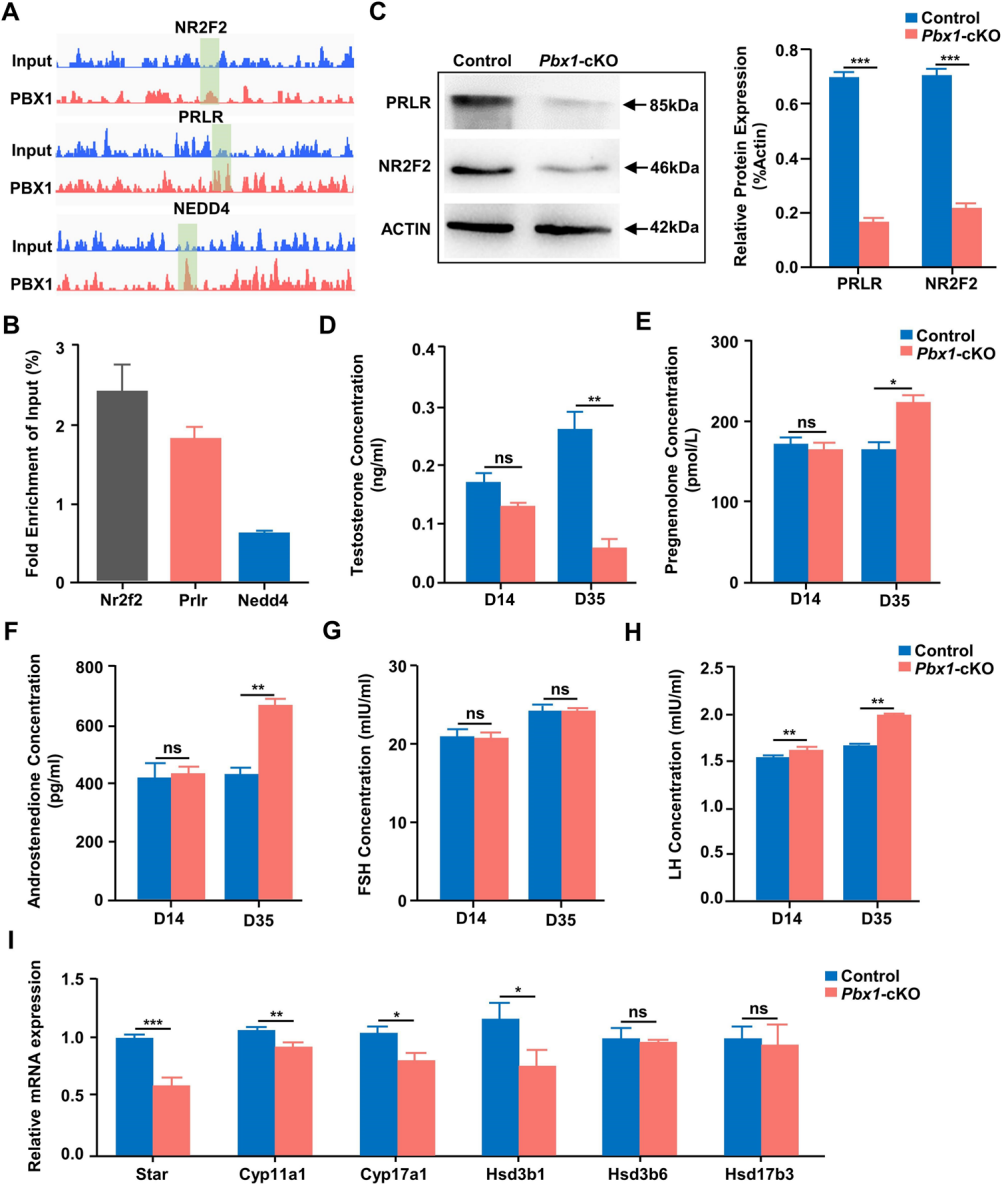
Transcription of *Nr2f2* and *Prlr* is directly regulated by *Pbx1* in Leydig cells. **A** IGV visualization of *Nr2f2*, *Prlr*, and *Nedd4* bound by *Pbx1* in the promoter regions. **B** ChIP‒qPCR validation of *Pbx1* binding sites relative to the input (*n* = 3). **C** Representative western blot images showing PRLR and NR2F2 expression in control and *Pbx1*-cKO testes (*n* = 3). The protein expression levels were calculated relative to those of beta-actin. **D** Testosterone hormone levels in control and *Pbx1*-cKO male mice (*n* = 3). **E** Testicular pregnenolone concentration in control and *Pbx1*-cKO male mice (*n* = 3). **F** Testicular aldrostenedione (ASD) concentration in control and *Pbx1*-cKO male mice (*n* = 3). **G** Follicle-stimulating hormone (FSH) assay in control and *Pbx1*-cKO male mice (*n* = 3). **H** Luteinizing hormone (LH) assay in control and *Pbx1*-cKO male mice (*n* = 3). **I** Relative abundances of transcripts involved in the process of testosterone synthesis determined via quantitative qRT‒PCR (*n* = 3). *, ** and *** denote significant differences at *P* < 0.05, *P* < 0.01, and *P* < 0.001, respectively; ns denotes not significant. The error bars represent the SDs

Next, we performed chromatin immunoprecipitation sequencing (ChIP-seq) analysis to determine the DNA sequences that are directly associated with *Pbx1*. After quality control, the genome-wide read density was calculated and normalized (RPM value) with a fixed window (5 kb) to pair-display read enrichment between the *Pbx1* antibody and input samples (Supplementary Fig. 8A–D). A total of 29,925 genes were bound by *Pbx1*, and 1186 genes were bound by *Pbx1* in the promoter region. GO enrichment and KEGG analyses revealed that the genes potentially regulated by *Pbx1* were enriched in G-protein coupled receptor and olfactory transduction signaling pathways (Supplementary Fig. 8E, Supplementary Table 6). Among these genes, *Nr2f2*, *Prlr*, *Nedd4*, *Fgl2*, *Phldb2*, *Cd63*, *Plxdc2*, *Dcn* and *Hsd17b12* were found to be downregulated in Leydig cells from *Pbx1*-cKO animals (Supplementary Table 5). The binding of *Pbx1* to the promoter regions of *Prlr*, *Nr2f2* and *Nedd4* were significantly enriched, which was validated by the ChIP–qPCR results (Fig. 8A, B). Western blotting analysis again confirmed that the expression of these genes was significantly decreased at the protein level in the *Pbx1* knockout testis (Fig. 8C).

Because genes directing Leydig cell development and testosterone production are dysregulated by *Pbx1* loss-of-function, we measured pregnenolone, androstenedione and testosterone concentrations in the testicular tissues of control and *Pbx1*-cKO animals. The testosterone concentration decreased by 76.92% at PD35 (0.06 ± 0.01 vs 0.26 ± 0.02 control, *n* = 3); strikingly, the pregnenolone concentration increased by 42.25% (229.30 ± 19.47 vs 161.20 ± 5.85 control, *n* = 3), and the androstenedione concentration increased by 48.89% (659.90 ± 23.81 vs 443.2 ± 16.18 control, *n* = 3) in the *Pbx1-*cKO mice compared with the control mice (Fig. 8D–F). The concentration of luteinizing hormone (LH) increased significantly in the testes of *Pbx1*-cKO mice at PD14 (1.62 ± 0.01 vs 1.48 ± 0.02 control, *n* = 3) and PD35 (1.98 ± 0.05 vs 1.67 ± 0.01 control, *n* = 3) (Fig. 8H). The concentration of follicle-stimulating hormone (FSH) did not differ (Fig. 8G). Moreover, we performed qRT‒PCR to investigate the relative expression of the genes involved in testosterone synthesis and found that the *Star*, *Cyp11a1*, *Cyp17a1* and *Hsd3b1* mRNAs were significantly lower in the *Pbx1*-cKO testes (0.61 ± 0.07 vs 1.00 ± 0.02, 0.94 ± 0.03 vs 1.07 ± 0.03, 0.81 ± 0.07 vs 1.04 ± 0.07, 0.77 ± 0.15 vs 1.17 ± 0.16 control, *n* = 3), whereas the expression of *Hsd3b6* and *Hsd17b3* was comparable between the control and *Pbx1*-cKO testes (Fig. 8I). Considering the major role of testosterone in maintaining the integrity of seminiferous tubules and the development of spermatids, we treated control and *Pbx1-*cKO mice with exogenous testosterone at a previously validated dose [38]. We supplemented the mice with testosterone for 14 days and examined the testis weight and histology of the seminiferous tubules. The serum testosterone concentration in *Pbx1*-cKO animals receiving the treatment (0.20 ng/mL) was restored to a comparable level as that in control mice (0.26 ng/mL); however, the testis weight remained low in the *Pbx1*-cKO animals, and spermatogenic defects persisted in the animals receiving testosterone supplementation (Supplementary Fig. 9). These data suggested that the production of testosterone and other hormones was affected by *Pbx1* deletion in Leydig cells; however, exogenous testosterone supplementation alone did not rescue the spermatogenic defects caused by *Pbx1* deletion.

## Discussion

Leydig cells are essential for testicular development and the maintenance of spermatogenesis. In the present study, we showed that the transcription factor PBX1 is exclusively expressed in Leydig cells and PTMCs in the testis. Conditional deletion of *Pbx1* altered the developmental trajectory of Leydig cells and altered the genetic program of testosterone biogenesis, which then caused problems in sustaining seminiferous tubules and spermatid differentiation. The findings of this study provide new insights into the molecular control of Leydig cell function and testosterone production.

PBX1 expression in the Leydig cell lineages and PTMCs in the testis throughout development. Leydig cells share a common origin with PTMCs, and perivascular cells in the fetal testis can give rise to these two cell populations [39]. FLCs produce high levels of androgen to direct sexual differentiation and the development of secondary sex characteristics [40]. The endocrine function of these cells is replaced by that of other androgen-producing cells in the postnatal testis [10]. Recent evidence from lineage tracing experiments indicates that adult and fetal Leydig cells are derived from *Wnt5a*-expressing cells [41]. These cells can form PTMC populations through an undefined mechanism. In the present study, we detected PBX1 expression in FLCs and ALCs as well as PTMCs in the neonatal and postnatal testes. A limited number of genes have been shown to exhibit similar expression patterns [42, 43]. For example, the transcription factor TCF21 marks multipotent progenitors that differentiate into Leydig cells and PTMCs [43]. These cells participate in early sex differentiation and contribute to testis regeneration after insult and homeostasis during aging [43, 44].

*Pbx1* in Leydig cells is essential for maintaining the integrity of seminiferous tubules in adult mice. Several transcription factors have been demonstrated to have pivotal functions in maintaining normal Leydig cell function. For example, depletion of SF-1 in steroidogenic cells causes a dramatic reduction in Leydig cell volume and impaired steroidogenesis [45]. The transcription factor GATA4 controls the expression of genes in the androgenic biogenesis pathway, and its knockdown in Leydig cells reduces pregnenolone, androstenedione and testosterone production [46]. Deletion of *Gata4* in Leydig cells and Sertoli cells causes testicular atrophy and defects in the motility and quantity of sperm [47]. In the present study, deletion of *Pbx1* not only caused a significant decrease in the sperm concentration but also resulted in damage to the basement membrane of the spermatogenic tubules, causing an influx of germ cells into the interstitial region.

Leydig cells interact with germ cells, Sertoli cells and peritubular myoid cells to maintain homeostasis of the seminiferous epithelium. Insufficient testosterone production and impaired Leydig cell function caused by *Pbx1* deletion led to disruption of the integrity of the seminiferous tubules, as evidenced by disorganization of the testicular structure and the appearance of spermatogenic cells in the interstitial space. This is an interesting observation because the deletion of androgen receptors in Sertoli cells or PTMCs does not cause a similar phenotype [48, 49]. Abnormalities in spermatogenic cells, including meiotic defects and impaired sperm function, are partially due to changes in the microenvironment. Meiotic and postmeiotic germ cells require a special environment separated by the blood‒testis barrier for development and differentiation [50]. We speculated that *Pbx1*-deficient Leydig cells fail to provide proper signals to guide other somatic cells, particularly Sertoli cells and PTMCs, which are the main contributors to the basement membrane of seminiferous tubules in the testis; therefore, these cells significantly affect meiosis and spermiogenesis.

Another important finding is that loss of *Pbx1* impaired Leydig cell function in adult testes. *Pbx1*-null mice fail to develop urogenital organs, which exhibit decreased proliferation and reduced expression of steroidogenic factor-1 (SF-1) in genital ridges [28]. PBX1 is essential for the formation and differentiation of multiple organs, and its mutation is associated with several diseases in humans [28, 51]. Notably, a de novo missense mutation in the TALE homeodomain of PBX1 is involved in human gonadal dysgenesis [30]. In this study, we found that PBX1 expression persists in the postnatal testis and that its conditional deletion in Leydig cells causes spermatogenic defects and sterility. In the present study, *Pbx1* was deleted in fetal Leydig cells around E12.5; however, the gonocyte-to-spermatogonia transition appeared to be normal, and spermatogenesis was initiated in the postnatal testis, indicating that *Pbx1* was dispensable for germ cells during the late fetal and neonatal periods of development. However, profound defects in testicular structure and spermatogenesis emerge in the postnatal testis after puberty. At least two abnormalities in Leydig cells were detected: dysregulated gene programs involved in testosterone synthesis and imbalanced differentiation of Leydig progenitor cells.

Leydig cells in the adult testis are a heterogeneous cellular population containing stem, progenitor, and mature cells. The absence of *Pbx1* changed the ratio of different subsets of Leydig cells. For example, the progenitor pool was decreased, with a particularly significant increase in ALCs. Although the origin and differentiation pathways of ALCs have been fully resolved, our scRNA-seq analysis of marker gene expression indicated that at least three different states existed in the Leydig cells and that *Pbx1* deletion changed the proportion of ALCs by 21.85%. Gene expression analysis demonstrated that transcription programs controlling lipid metabolism were dysregulated by *Pbx1* deletion in ALCs. *Pbx1* regulated the expression of genes encoding enzymes involved in androgen biogenesis, including *Nr2f2* and *Prlr*. *Nr2f2* is a nuclear receptor that plays a pivotal role in directing the formation of functional steroidogenic ALCs [52]. A mutation in the human NR2F2 gene causes impairments in testis and cardiac function [18]. *Nr2f2* regulates steroidogenesis by directly promoting *Insl3* and *Star* expression [53, 54]. Therefore, we speculate that the decreased testosterone synthesis in *Pbx1*-cKO mice was likely due to the reduced expression of *Star* rather than the lack of *Hsd17b3*, which is responsible for transporting cholesterol into the mitochondria [55]*. Prlr* belongs to the class I cytokine receptor family; it is a receptor for prolactin and is expressed in a variety of tissues and organs in mammals [56]. The prolactin receptor is fully functional in Leydig cells, and its activation stimulates testosterone synthesis in a dose-dependent manner [57].

Decreased testosterone is not the sole cause of spermatogenic failure in the adult testis. We treated *Pbx1*-cKO mice with exogenous testosterone for 14 days and examined the possibility that testosterone supplementation rescues the impairments in seminiferous tubule and spermatid development. During the treatment, although testosterone returned to the normal level, the testicular androstenedione and pregnenolone levels were still lower, and the spermatogenic defects were not rescued by exogenous testosterone supplementation in the *Pbx1*-cKO mice. In humans, decreased testosterone levels lead to gonadal dysfunction and are associated with cardiovascular disease, aging and chronic health diseases [58]. Whether *Pbx1*-deficient animals develop other health problems awaits further investigation.

In conclusion, the transcription factor PBX1 is expressed in Leydig cells and PTMCs in the testis, and its conditional deletion causes sterility due to profound defects in spermatogenesis. Mechanistically, *Pbx1* directly binds to genes involved in testosterone biogenesis, and its loss of function results in the downregulation of *Prlr*, *Nr2f2* and other genes, which causes the accumulation of pregnenolone and a decrease in testosterone. The deletion of *Pbx1* mainly affects the anterior part of the testosterone synthesis process and is not caused by changes in the number of Leydig cells or the deletion of *Hsd17b3*. Gene expression and developmental trajectory analyses indicated that *Pbx1* was also needed for proper differentiation of Leydig progenitor cells.

## Materials and methods

### Animals

All animal experiments were performed according to the Guide for the Care and Use of Laboratory Animals and were approved by the Animal Welfare and Ethics Committee at the Northwest Institute, Chinese Academy of Science. *Pbx1*^*flox/flox*^ mice and *Cyp17a1-Cre* (C001049, C57BL/6J) mice were generated by using CRISPR‒Cas9 technology (from the Cyagen Company). *Pbx1*^*flox/flox*^ females were mated with *Cyp17a1-Cre*^+^ males to generate *Cyp17a1-Cre*^+^*; Pbx1*^*flox/*^ + male mice. *Cyp17a1-Cre*^+^*; Pbx1*^*flox/*+^ male mice were mated with *Pbx1*^*flox/flox*^ mice to generate *Cyp17a1-Cre*^+^*; Pbx1*^*flox/flox*^ (*Pbx1*-cKO) male mice. *Cyp17a1-Cre*^+^*; Pbx1*^*flox/*+^ mice were used as controls. All mice were on a 129S2/SvPasCrl; C57BL6N mixed background. For the testosterone treatment, adult male *Pbx1*-cKO mice were subcutaneously injected with testosterone (3.7 g/kg) as described previously [38]. Simultaneously inject equal volumes of corn oil (Sigma, Lot # 66633) into the control mice. After 14 days, testicular tissue was taken and morphological analyses were performed. At least three control and knockout animals were used for each experiment. The primers used for genotyping and all reagents used for all the experiments are listed in Table S1.

### Fertility test and sperm analysis

*CYP17a1-Cre*^+^*; Pbx1*^*flox/*+^ male mice at 35 days of age were paired with four adult females for 4 months, and the average litter size was recorded every month as previously described [59]. To measure the sperm concentration, the cauda epididymis was cut, and the spermatozaoa were squeezed from the cauda epididymis with small scissors in 1 mL of human tubal fluid (HTF) (Merck Millipore, MA, USA). The samples were then kept for 10 min at 37 °C to release sperm. The harvested sperm were diluted, and the sperm concentration was determined by using a computer-assisted sperm analysis system. Approximately 500 µL of supernatant was aspirated, and 0.5 µL of PNA and 0.5 µL of MitoTracker were added. After incubation at 37 °C for 2 h, the samples were stained with 200 µL of H33342 for 30 s. The slides were examined under a fluorescence microscope (Lecia, Germany).

### Histology and immunohistochemical staining

Immunohistochemical (IHC) and immunofluorescent (IF) staining of testicular samples was conducted as described previously [60]. Briefly, testicular tissues were fixed in Bouin’s solution or 4% paraformaldehyde (PFA) and embedded in paraffin. Tissues were cut into 4 µm sections, and cross-sections of testicular samples were rehydrated and stained with hematoxylin and eosin (H&E). For immunostaining, after antigen retrieval, endogenous peroxidase activity was blocked by 3% H_2_O_2_ for 10 min at room temperature (RT). Nonspecific binding was blocked, and the sections were incubated with primary antibody overnight at 4 °C. Normal IgG served as a negative control. The samples were then incubated with the secondary antibody for 2 h at RT. The sections were stained with H33342 (IF) or 3,3′-diaminobenzidine tetrachloride (DAB) solution (IHC). The cells were observed, and images were captured using a microscope (Leica, TELLARIS 5 SR). The antibodies used in the study are listed in Table S1.

### Isolation and culture of Leydig cells

The testes were collected and placed in cold Dulbecco’s PBS (DPBS), and the Leydig cells were isolated as described previously [61]. The cells were collected in DMEM/F12 (containing 10% FBS) and incubated for 12 h at 34 °C in a 5% CO_2_ incubator. Subsequently, the cells were collected and centrifuged at 600*g* for 6 min, the supernatant was discarded, and the cells were resuspended in DMEM/F12 (containing 10% FBS). After washing, the cells were collected and subjected to single-cell transcriptome sequencing.

### Single-cell transcriptome analysis

Leydig cell-enriched single-cell suspensions were centrifuged in 30% Percoll (Sigma, USA) in DPBS-S to remove Sertoli cells. Red blood cells were removed using Red Blood Cell Lysis Buffer (Solarbio, China). Single-cell capture and cDNA synthesis were conducted on a BD Rhapsody™ Single-Cell Analysis System (Doc ID: 210966 Rev.1.0; PROTOCOL) to capture single cells and synthesize cDNA. Library preparation was carried out according to the BD Rhapsody™ System mRNA Whole Transcriptome Analysis (WTA) and AbSeq Library Preparation Protocol. Paired-end sequencing (150 bp) was performed on the Illumina HiSeq 2000 platform (sequenced by Personalbio).

Quality control and analysis of the raw data were conducted according to the BD Rhapsody pipeline. Pipeline filtering and removal of low-quality reads were performed according to the following criteria: read1, length < 66 bp; read2, length < 64 bp; mean base quality score < 20 for read1/read2; and read 1, highest single nucleotide frequency (SNF) ≥ 0.55 or read2 of SNF ≥ 0.80. Reader 1 included a cell label containing three sections and two linker sequences, a unique molecular index (UMI) including eight random bases, and a poly(T) tail. A read1 had to have three components for it to be retained: a perfect match to a predefined cell label sequence at expected locations, a UMI with non-N bases, and at least 6 T in the 8 bases following UMI. Read 2 was aligned to the mm10 mouse transcriptome (UCSC) using Bowtie 2 (V2.1.0). The following criteria were used for a given read2: the read was uniquely aligned to a gene sequence in the reference; the alignment began within the first 5 bases; the length of the alignment that could be a match or mismatch in the CIGAR string was > 60; and the read did not align to phiX174. The pipeline subsequently combined information from read 1 and read 2. Reads with the same cell label, same UMI sequence, and same gene were collapsed into a single raw molecule. To reduce the impact of PCR and sequencing errors on the number of raw molecules, the BD Phapsody pipeline used recursive substitution error correction (RSEC) to correct errors caused by base substitutions and distribution-based error correction (DBEC) to adjust for errors derived from library preparation steps or sequencing base deletions. The BD pipeline identifies the number of cells based on the second-order import algorithm. The cell-gene expression matrix file produced by the BD Phapsody pipeline was imported into R, and we used the Seurat (V4.4.0) package to conduct quality control, cell selection, data normalization, variable gene analysis, PCA-based dimensional reduction, clustering of the cells, data integration, DEG identification, and functional analysis of the DEGs. The monocle2 (V2.30.0) package was used to analyze the pseudotime, and differential gene–protein interaction (DEG) networks were mapped using the Search Tool for the Retrieval of Interacting Genes/Proteins (STRING) (V12.0) and Cytoscape (V3.9.1) software. The CellChat (V1.6.1) package was used to analyze cell-to-cell interactions.

### Hormone assays

Testicular tissue from different periods was taken at the same weight, ground in 500 µL of 1 × PBS, and centrifuged at 12,000*g* for 5 min, after which the supernatant was aspirated for testosterone concentration measurement. The testosterone concentration in testicular tissue was measured by a testosterone ELISA kit (Elabscience, E-EL-0155c), a pregnenolone ELISA kit (Elisalab, JYM0845Mo), an epistenedione (ASD) ELISA kit (Elisalab, JYM0371Mo), an FSH ELISA kit (Mbbiology, MB-6096A), and an LH ELISA kit (Mbbiology, MB-3318A) according to the manufacturer's instructions. The minimum detectable concentrations were 0.31 ng/mL, 10 pmol/L, 20 pg/mL, 2.5–80 mIU/mL, and 0.2–12 mIU/mL.

### Quantitative real-time reverse transcription PCR

Total RNA was extracted from mouse Leydig cells using an RNA simple Total RNA Kit (TIANGEN), and cDNA was synthesized from 200 ng of RNA using TranScript One-seq gDNA removal and cDNA Synthesis SuperMix (TtansGen Biotech). Real-time PCR was performed on an Applied Biosystems Real-Time PCR Setection System using PerfectStart Green qPCR SuperMix (TtansGen Biotech). Each PCR mixture consisted of 5 μL of PerfectStart Green qPCR SuperMix, 2 μL of cDNA sample, 2.6 μL of water and 0.2 μL of the gene-specific primers listed in Table S1. DNA fragment amplification was performed using the following thermal cycling program: 3 min at 95 °C, followed by 40 cycles of 15 s at 95 °C. 30 s at 63 °C and 30 s at 72 °C. Relative mRNA levels were determined with the ΔΔCt method using Gapdh as the internal reference gene.

### ChIP-seq and ChIP-qPCR

Testes were collected from wild-type mice, and testicular tissue was digested using collagenase (1 mg/mL) and DNase I (7 mg/mL) to obtain a single-cell suspension. The cells were crosslinked, and the chromatin was fragmented by sonication in lysis buffer [62]. The cross-linked chromatin was incubated with the indicated antibodies (anti-Pbx1, Proteintech, 18204-1-AP) in lysis buffer supplemented with RNase A (20 mg/mL) and Protease K. Protein G/magnetic beads were used to obtain chromatin‒protein complexes, which were washed with cold RIPA buffer (50 mM HEPES, 500 mM LiCl, 1 mM EDTA, 1% NP-40, 0.7% Na deoxycholate in ddH_2_O). After washing in TE buffer/50 mM NaCl, the product was dissolved in 210 µL of elution buffer (50 mM Tris, 10 mM EDTA, 1% SDS in ddH_2_O) in ddH_2_O for 15 min at 65 °C and treated with both RNaseA and proteinase K. After uncrosslinking, the ChIP DNA was purified by a microsample genomic DNA extraction kit (Tiangen, #DP316). Immunoprecipitated DNA was processed for deep sequencing (IIIumina HiSeq 2000, sequenced by Anoroad) or analyzed by qPCR (TransGen Biotech, AQ601-02) with promoter-specific primers.

To analyze the ChIP-seq data, PBX1 ChIP and input chromatin reads were aligned to the GRCm38.90 mouse genome with Bowtie2 (V2.1.0). The genomic regions were segmented as 2K upstream of the genes (11/25 Upstream2k), UTR5, exons (Exon), introns (Intron), UTR3, 2K downstream (Downstream2k), or intergenic regions (Intergenic). We used MACS2 (V2.1.1) software to predict the insert lengths of the samples. MACS2 scans the genome with a window of a certain size, counts the enrichment of reads in each window, and then draws 1000 windows as samples to construct enrichment models to predict the average insert length, which shows that the bimodal model of the sample has obvious peaks and a sharp morphology. Only uniquely mapped tags on the mouse genome were retained. Peaks were identified with MACS2, and the bedfile produced was annotated using ChIPseeker (V1.38.0) in R. The sequence of DNA fragments bound by *Pbx1* was analyzed with MEME (V4.12.0). Then, we designed primers specific for the *Pbx1*-bound regions and used PCR to determine the enrichment of PBX1 in the promoter regions of these genes. Input and normal IgG were used as positive and negative controls, respectively.

### Transmission electron microscopy of testes

Testicular tissues were prefixed with 3% glutaraldehyde, postfixed in 1% osmium tetroxide, dehydrated in a series of acetone solutions, infiltrated with Epox 812, and embedded. The semithin sections were stained with methylene blue, and ultrathin sections were cut with a diamond knife and stained with uranyl acetate and lead citrate. Sections were examined with a JEM-1400-FLASH transmission electron microscope.

### Chromosome spreading

Seminiferous tubules were dispersed and treated in hypotonic solution (30 mM Tris–Cl, 50 mM sucrose, 17 mM sodium citrate, 5 mM EDTA in ddH_2_O) for 20 min. A 1 mM sucrose working solution was used to release cells from the seminiferous tubules. The cell suspension was evenly spread on adhesive slides soaked in 1% PFA and placed in a hot and wet box overnight. The cells were washed twice with 0.2% Kodak Professional Photo-Flo 200 and then placed in ADB for 1 h after drying and drying at RT. The primary antibody was diluted with ADB, added to the slides, which were subsequently sealed with rubber cement and incubated at 37 °C for 24 h. After removing the cement, the slides were immersed in ADB for 30 min to automatically detach the cover glass. The cells were soaked in ADB for 1.5 h and dried, after which the secondary antibody was added and incubated at 37 °C for 24 h. After the samples were washed with ADB for 30 min and PBS for 1 h, the slices were stained with H33342, mounted in 50% glycerin, and examined under a fluorescence microscope (Lecia, Germany). The reagents used are listed in Supplementary Table 2.

### Western blot

Total testicular protein was extracted from the testes of control and *Pbx1*-cKO mice using the KeyGEN Biological Whole Protein Extraction Kit (No. KGP 2100). The protein concentration was determined by a BCA protein assay kit (Lot: 17D01A46), and the proteins were denatured at 100 °C for 5 min before western blotting. An SDS‒PAGE gel preparation kit was used to prepare a 10% separating gel and a 5% stacking gel. The proteins were transferred to PVDF membranes, and nonspecific binding was blocked with 5% skim milk powder. Beta-ACTIN, KIT, NR2F2, PRLR, CXCL12 and WNT5A/B antibodies were prepared with 5% skim milk and incubated at 4 °C for 16 h, followed by three washes with 1 × TBST. The secondary antibodies HRP-conjugated goat anti-rabbit IgG and goat anti-mouse IgG were prepared with 5% skim milk, incubated at RT for 2 h and washed three times with 1 × TBST, followed by exposure to visualize the results. The reagents used are listed in Supplement Table S2.

### Statistical analysis

Assessment of statistical significance was performed using a two-tailed unpaired *t test* for normally distributed data. All the statistical analyses were performed using GraphPad Prism v7. The statistical significance of the differences is expressed as follows: *P < 0.05, **P < 0.01, ***P < 0.001, and ****P < 0.0001.

### Supplementary Information

Below is the link to the electronic supplementary material.

Supplementary file1 (DOCX 4462 KB)

Supplementary file2 (XLSX 13 KB)

Supplementary file3 (XLSX 29 KB)

Supplementary file4 (XLSX 15 KB)

Supplementary file5 (XLSX 13 KB)

Supplementary file6 (XLSX 1311 KB)

Supplementary file7 (XLSX 17 KB)

## Data Availability

All the data supporting the findings of this study are available from the corresponding author upon reasonable request.

## Abbreviations

FLCs: Fetal Leydig cells
ALCs: Adult Leydig cells
SLCs: Stem Leydig cells
PLCs: Progenitor Leydig cells
*Pbx1*-cKO: Cyp17a1-Cre^+^; Pbx1^flox/flox^ mice
HPG: Hypothalamic–pituitary–gonadal
PTMCs: Peritubular myoid cells
SSCs: Spermatogonial stem cells
DSBs: DNA double-strand breaks
scRNA-seq: Single-cell RNA-seq
UMIs: Unique molecular index
UMAP: Uniform manifold approximation and projection
DEGs: Differentially expressed genes
TEM: Transmission electron microscopy
FSH: Follicle-stimulating hormone
LH: Luteinizing hormone
LHR: Luteinizing hormone receptor
HTF: Human tubal fluid
TUNEL: TdT-mediated dUTP nick-end labeling
