# Supplementary material for: Loss of PBX1 function in Leydig cells causes testicular dysgenesis and male sterility
Source: Cell Mol Life Sci. 2024 May 9;81(1):212. doi: 10.1007/s00018-024-05249-5 (PMC11082031; doi:10.1007/s00018-024-05249-5)
Supplement: Supplementary file 1 — Supplementary file1 (DOCX 4462 KB) [file 18_2024_5249_MOESM1_ESM.docx]

**Supplementary Materials**

**Loss of PBX1 Function in Leydig Cells Causes Testicular Dysgenesis and Male Sterility**

**Authors**: Fei-Chen Wang^1,2,#^, Xiao-Na Zhang^1,2,#^, Shi-Xin Wu^1,2^, Zhen He^1,2^, Lu-Yao Zhang^1,2,3^, Qi-En Yang^1,2,3,*^

**Affiliations:** ^1^Key Laboratory of Adaptation and Evolution of Plateau Biota, Northwest Institute of Plateau Biology, Chinese Academy of Sciences, Xining, 810001, Qinghai, China; ^2^University of Chinese Academy of Sciences, Beijing, 100049, China; ^3^Qinghai Provincial Key Laboratory of Animal Ecological Genomics, Northwest Institute of Plateau Biology, Chinese Academy of Sciences, Xining, 810001, Qinghai, China.

^#^These authors contributed equally.

***Corresponding author**

**Q.E. Yang**

Address: Qinghai Provincial Key Laboratory of Animal Ecological Genomics, Northwest Institute of Plateau Biology, Chinese Academy of Sciences, Xining, Qinghai 810000, China

Tel: +86-971 15897112345

Email address: yangqien@nwipb.cas.cn

**
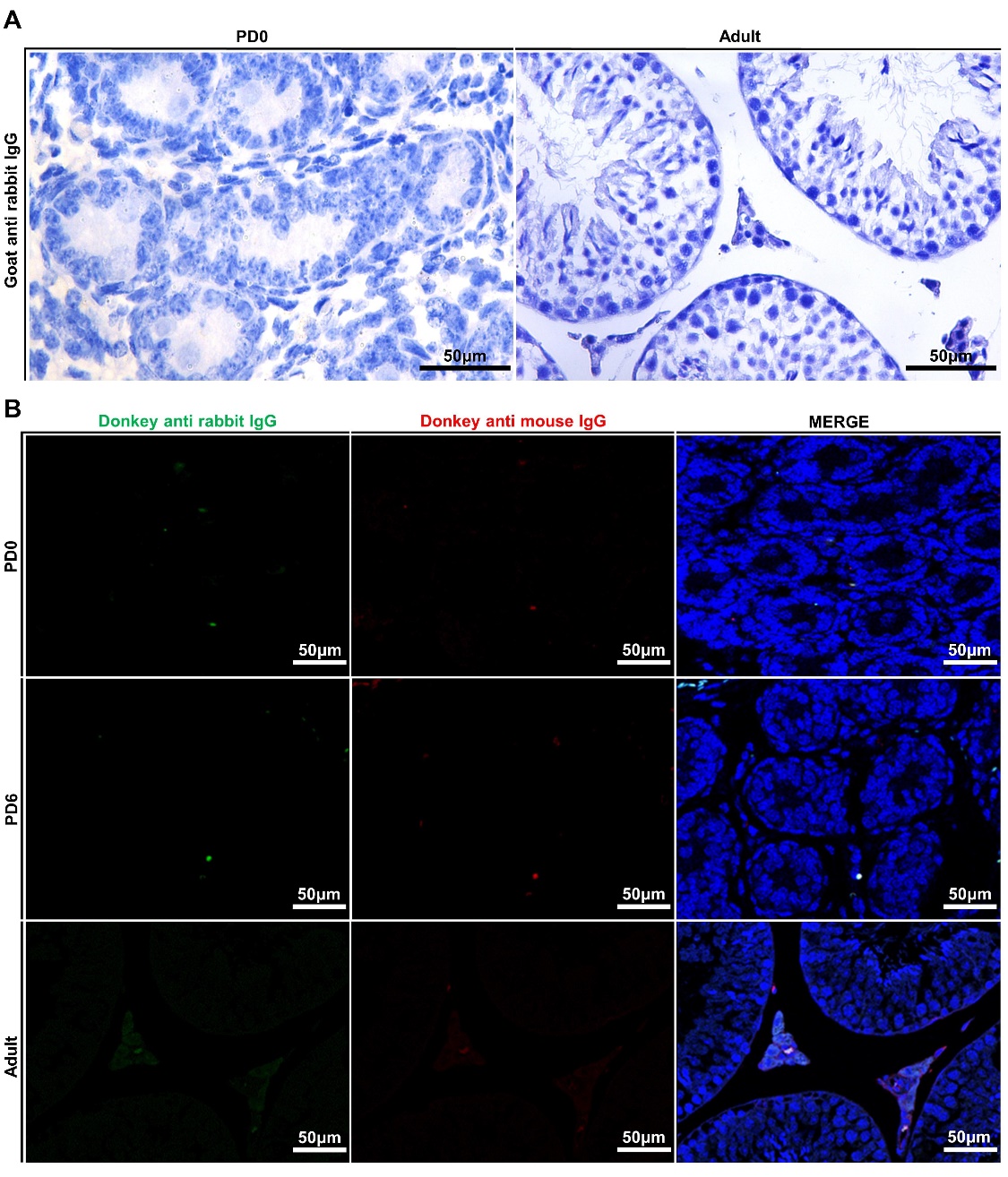
Supplementary Fig. 1**

**Supplementary Fig. 1 Negative controls for immunohistochemistry experiments.** (A) Negative controls for immunohistochemical staining of cross-sections from testes of newborn and adult male mice. Scale bar = 50 µm. (B) Negative controls for immunofluorescence staining of testicular cross-sections from controls at postnatal day (PD) 0, PD6 and PD60. Scale bar = 50 µm.

**
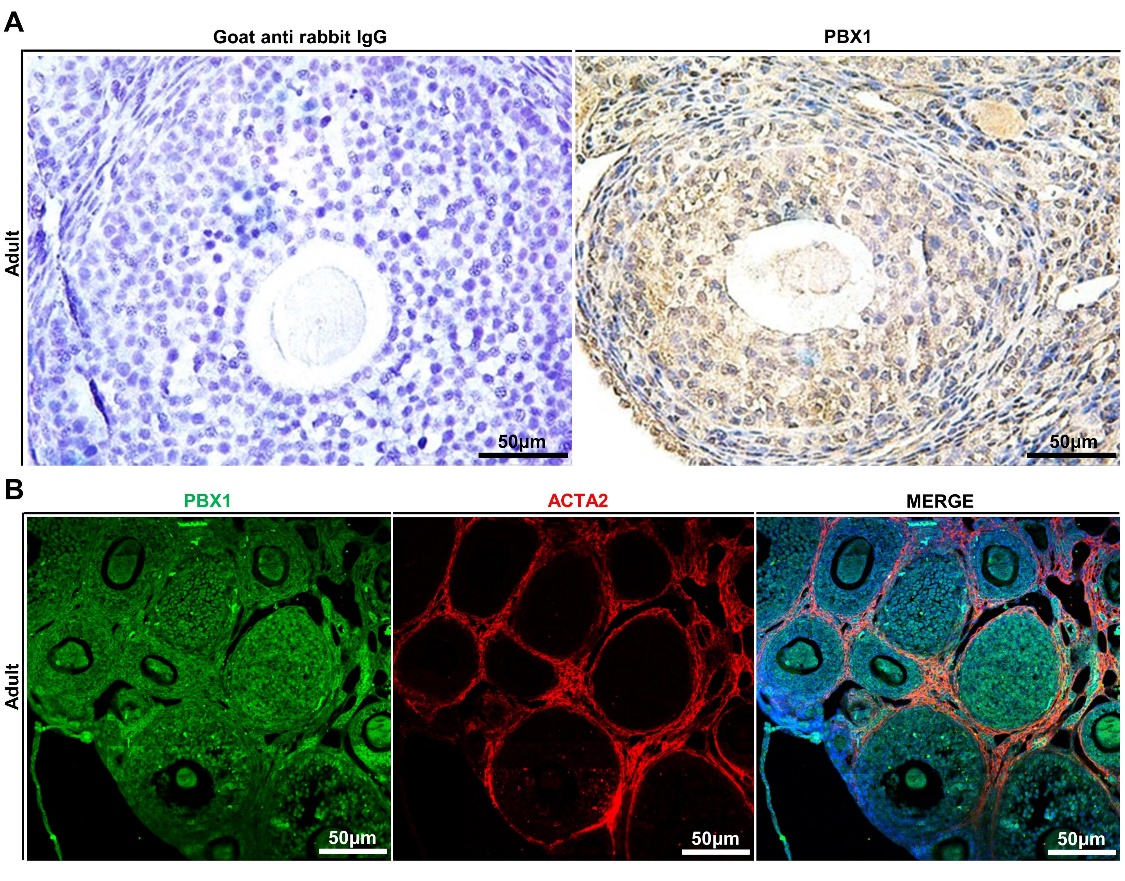
 Supplementary Fig. 2**

**Supplementary Fig. 2 PBX1 is widely expressed in the adult mouse ovary. (A)** Immunohistochemical staining of PBX1 in ovary cross-sections of wild-type adult mice (2 months old). Scale bar = 50 µm. (B) Immunofluorescence costaining of PBX1 (green) and ACTA2 (red) in ovary cross-sections of wild-type mice at PD60. Scale bar = 50 µm.

**
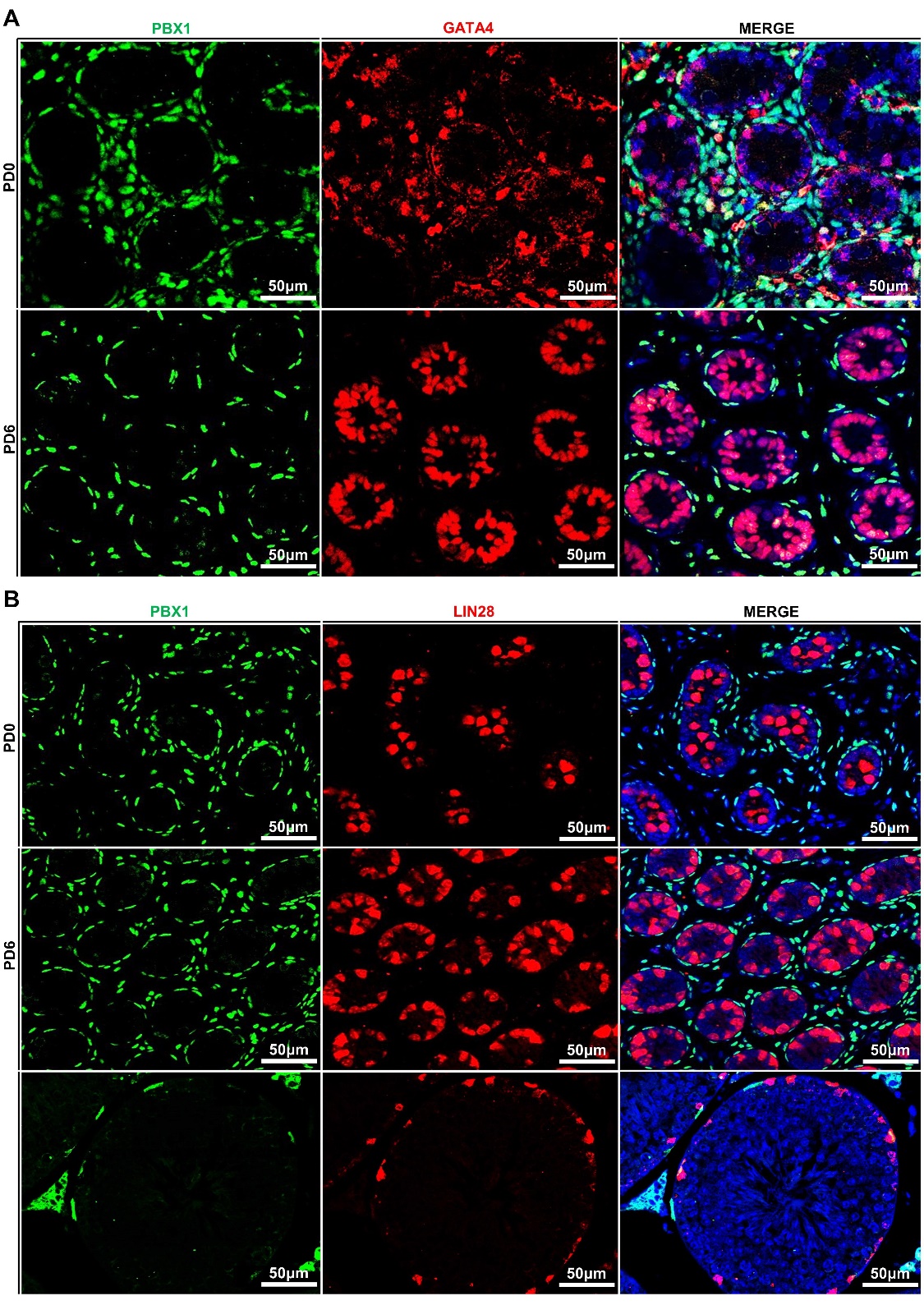
Supplementary Fig. 3**

**Supplementary Fig. 3 PBX1 expression is not detected in spermatogonia or Sertoli cells.** (A) Immunofluorescence staining of GATA4 (red) and PBX1 (green) in cross-sections of testes from PD0 and PD6 male mice. Scale bar = 50 µm. (B) Immunofluorescence staining of PBX1 (green) and LIN28 (red) in cross-sections of testes from mice at PD0, PD6 and Adult. Scale bar = 50 µm.

**
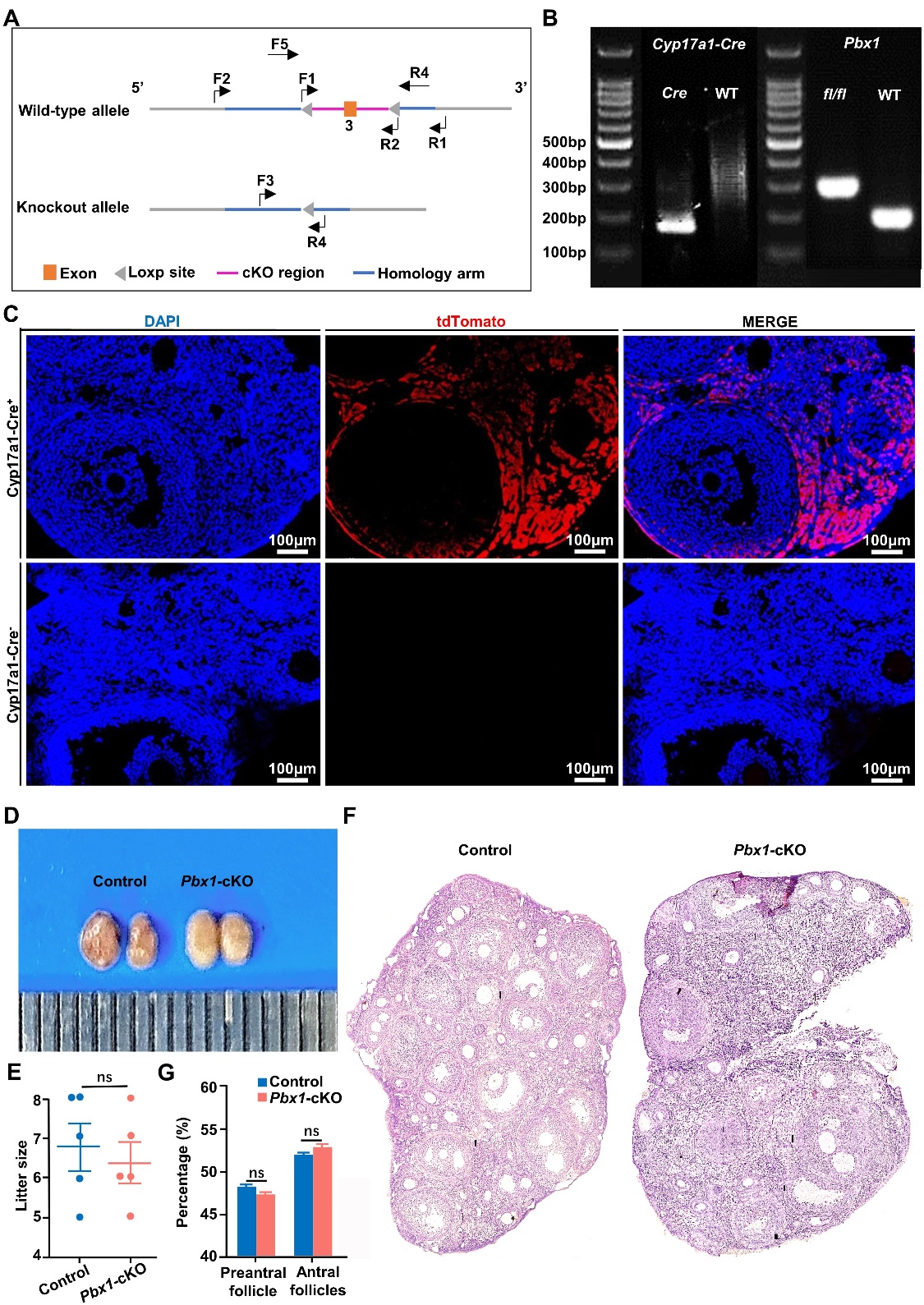
Supplementary Fig. 4**

**Supplementary Fig. 4 Conditional knockout of *Pbx1* in Cyp17^+^ cells in males and females using the Cre–LoxP system.** (A) Schematic diagram showing the strategy used to knock out the *Pbx1* gene. (B) Identification of the *CYP17a1-Cre* and *Pbx1* genes by gel electrophoresis. (C) Immunofluorescent costaining for tdTomato (red) and DAPI (blue) in cross-sections of testes from Cyp17a1-iCre [KI/+]; Rosa26-LSL-tdTomato [CKI/+] and control ovaries. (D) Representative images of testes from 4-month-old control and *Pbx1*-cKO female mice. (E) Litter sizes of control (n=5) and *Pbx1*-cKO female mice (n=5). (F) H&E-stained testes of control and *Pbx1*-cKO mice at 4 months of age. (G) Proportion of preantral and antral follicles in control and *Pbx1*-cKO mice (n=3).

**
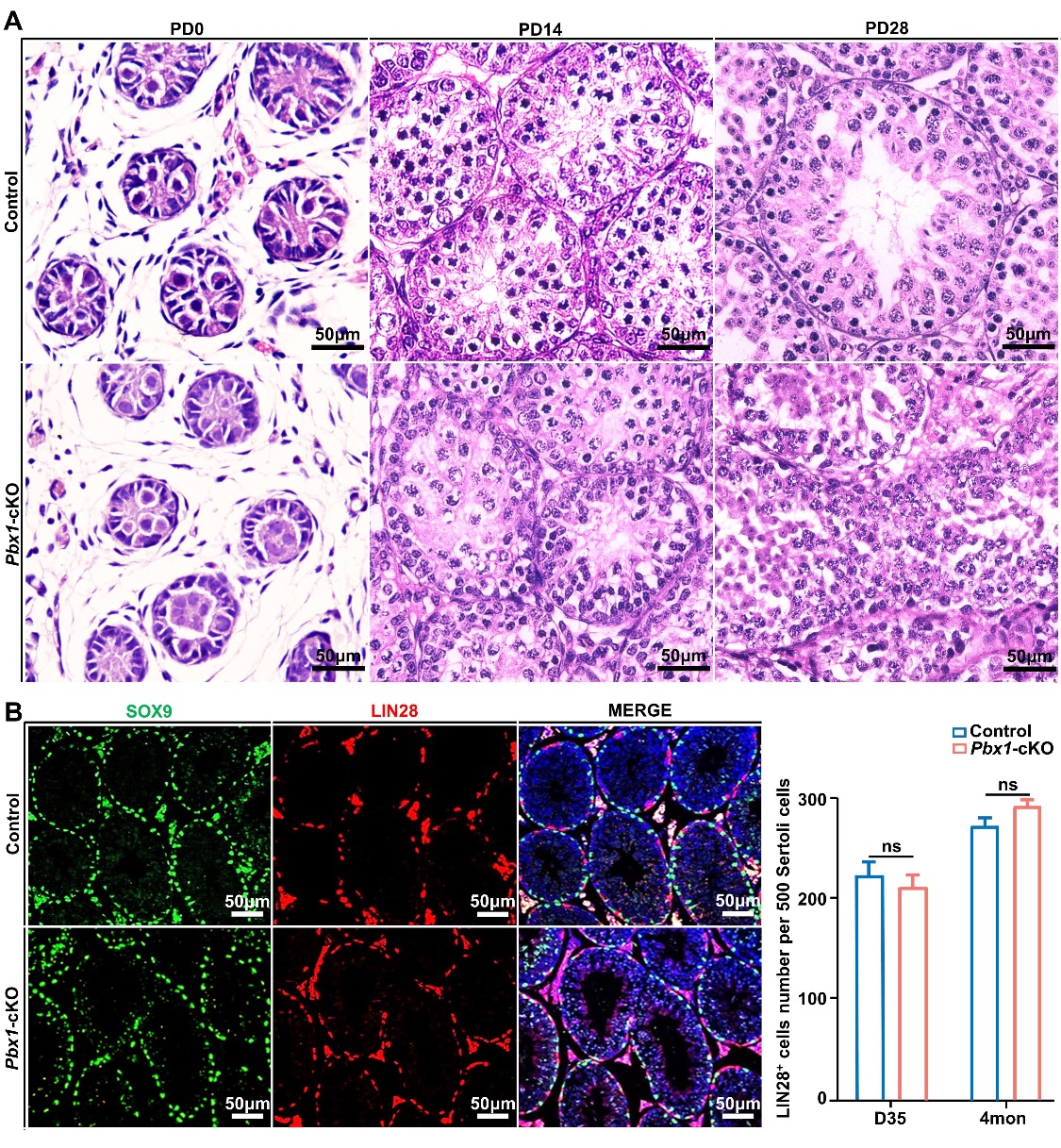
Supplementary Fig. 5**

**Supplementary Fig.** **5 *Pbx1* deletion in Leydig cells did not affect spermatogenic cell development in the neonatal testis.** (A) H&E-stained testicular cross-sections of control and *Pbx1*-cKO male mice at postnatal day (PD)0, PD14 and PD28. Scale bar = 50 µm. (B) Immunofluorescence staining of LIN28 (red) and SOX9 (green) in cross-sections of testes from 4-month-old male mice and the proportion of LIN28^+^ cells among every 500 Sertoli cells. ns denotes not significant. Scale bar = 50 µm.

**
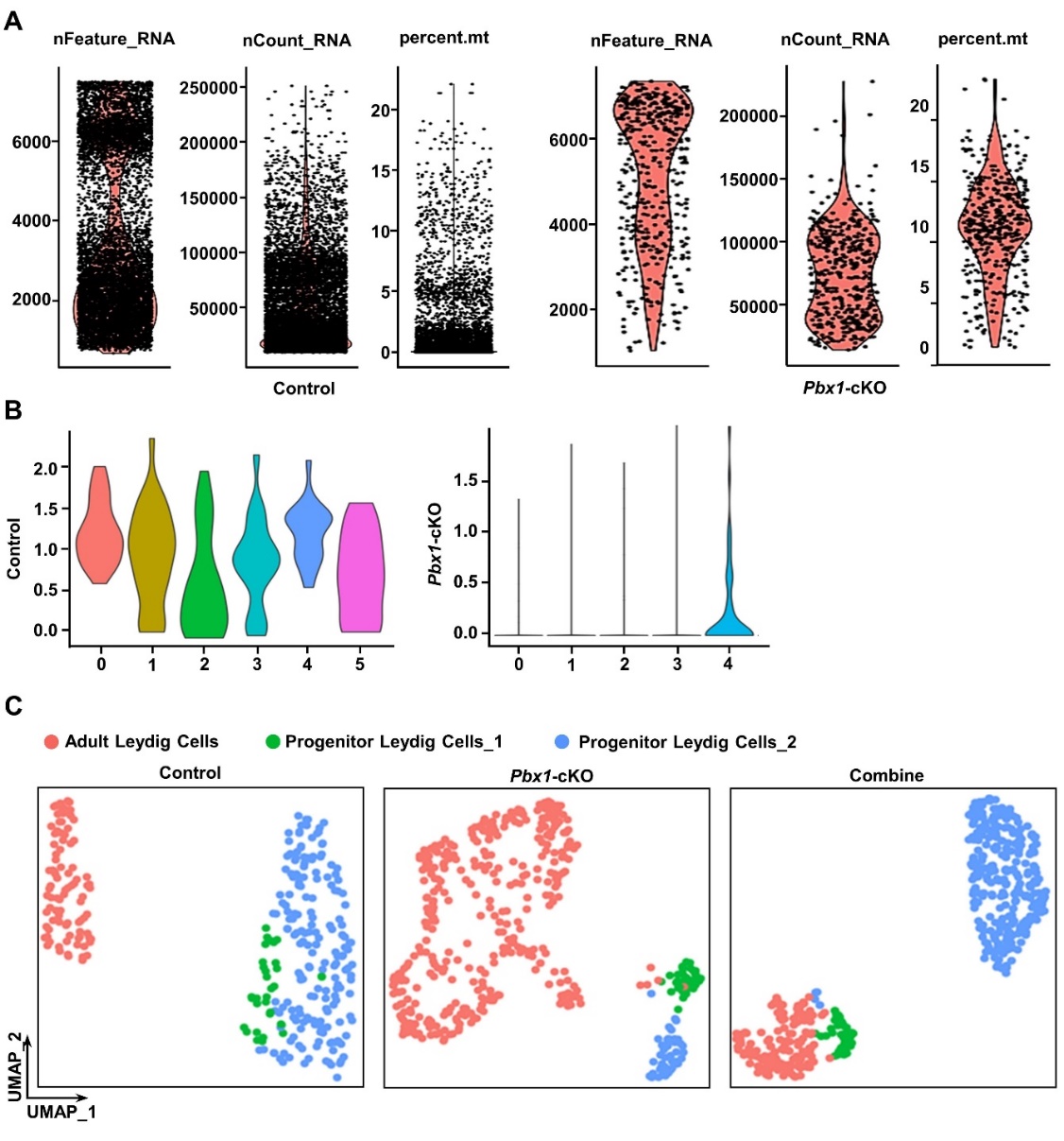
Supplementary Fig. 6**

**Supplementary Fig. 6 Quality control QC filtering and dimensionality reduction clustering of the scRNA-seq data.** (A) Gene number, count, and mitochondrial gene ratio of each cell according to the scRNA-seq data of the control and *Pbx1*-cKO samples. (B) Violin plot of *Pbx1* expression in Leydig cells from control and *Pbx1*-cKO mice. (C) Unbiased dynamic lineage analysis (UMAP) of Leydig cells extracted from the testes of control and *Pbx1*-cKO mice at 2-months of age (n=2).


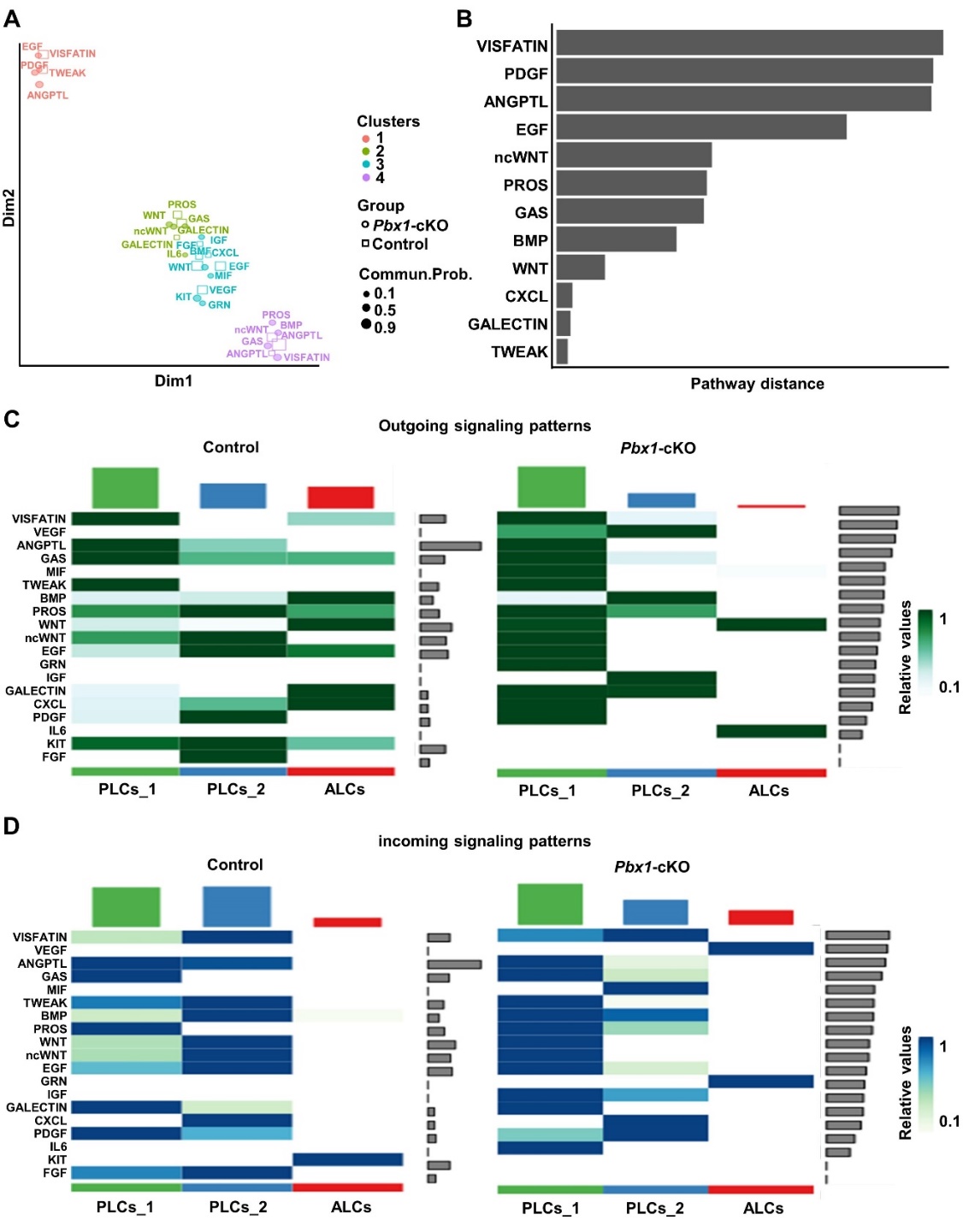
**Supplementary Fig. 7**

**Supplementary Fig. 7 Defective communication between different Leydig cell subtypes.** (A) Identification of signal groups based on structural similarity. (B) Calculated and visualized pathway distances. (C) Comparison of outgoing signaling patterns associated with each cell population. (D) Comparison of incoming signaling patterns associated with each cell population.


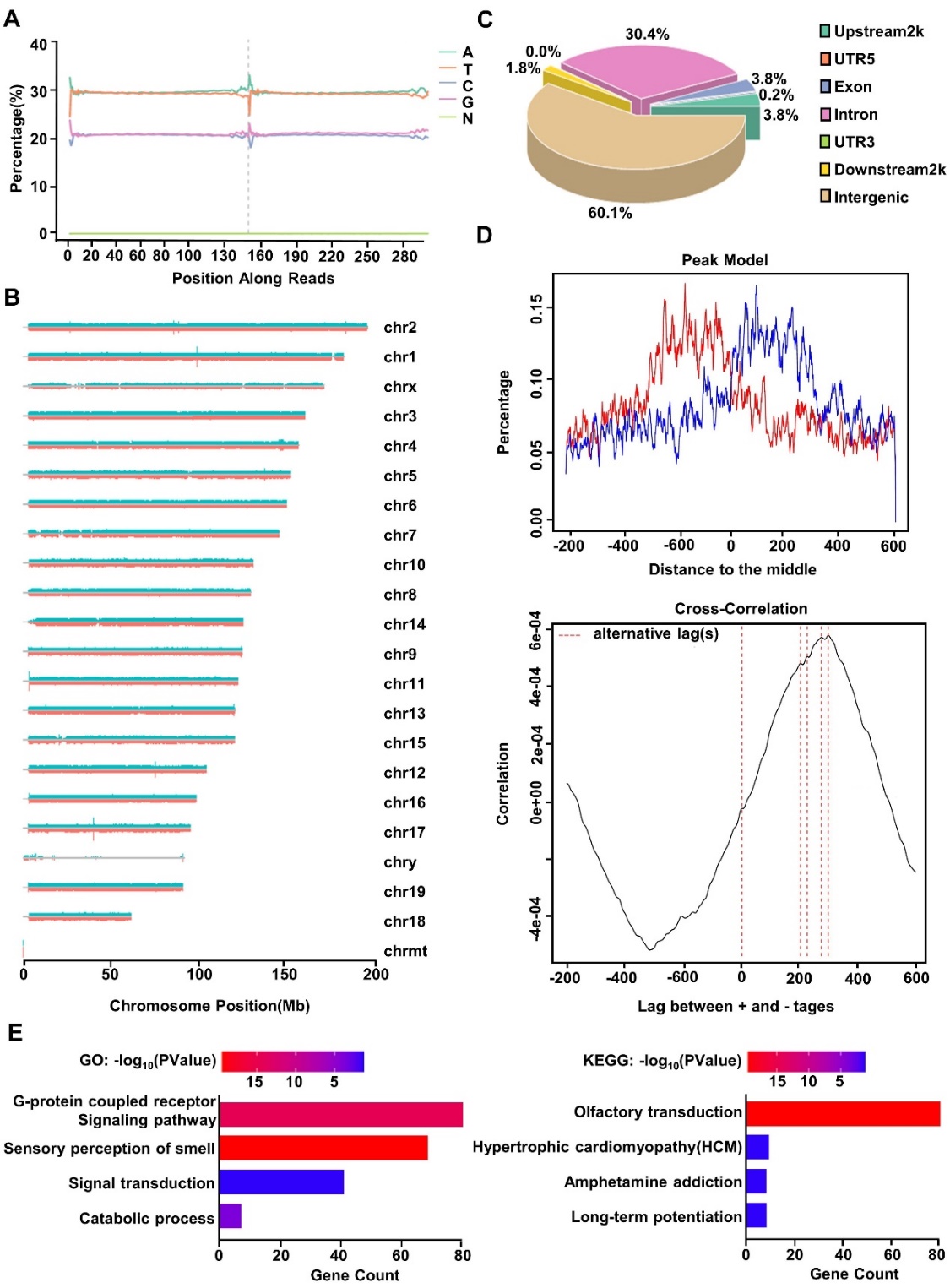
**Supplementary Fig. 8**

**Supplementary Fig. 8 Quality control and analysis of *Pbx1* Chip-seq data.** (A) Base distribution map of the reads obtained by sequencing. (B) Enrichment of reads on chromosomes between *Pbx1* ChIP and the input. (C) The distribution of peaks in genomic regions. (D) Length estimation distribution. (E) GO enrichment and KEGG pathway histogram plots for several promoter region-binding genes of *Pbx1*.

**
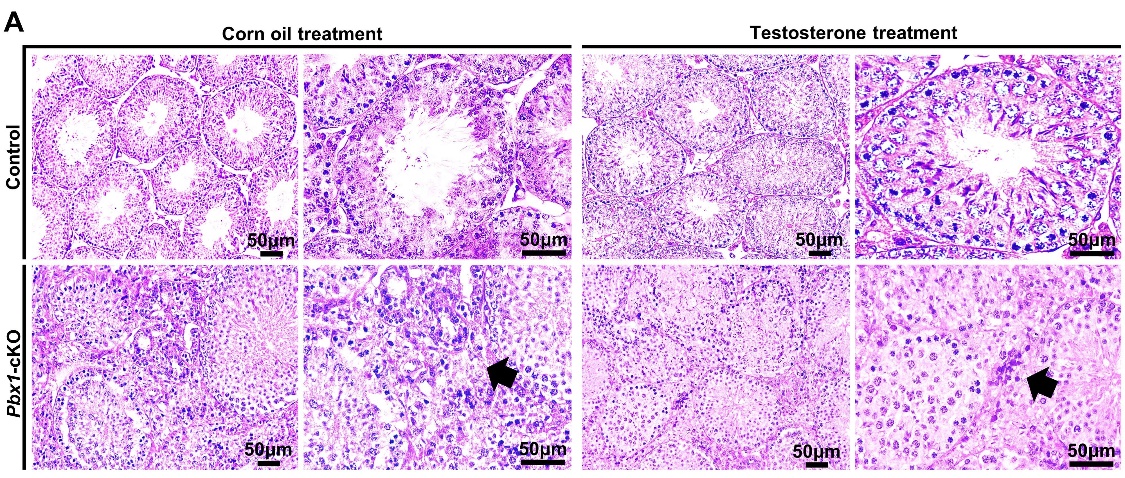
Supplementary Fig. 9**

**Supplementary Fig. 9 Supplementation with exogenous testosterone failed to reverse the spermatogenic defects caused by the loss of *Pbx1* in Leydig cells.** (A) HE staining of control and *pbx1*-cKO adult mice supplemented with equal doses of exogenous testosterone and corn oil. Scale bar = 50 µm.
